# Estimating cholera incidence with cross-sectional serology

**DOI:** 10.1126/scitranslmed.aau6242

**Published:** 2019-02-20

**Authors:** Andrew S. Azman, Justin Lessler, Francisco J. Luquero, Taufiqur Rahman Bhuiyan, Ashraful Islam Khan, Fahima Chowdhury, Alamgir Kabir, Marc Gurwith, Ana A. Weil, Jason B. Harris, Stephen B. Calderwood, Edward T. Ryan, Firdausi Qadri, Daniel T. Leung

**Affiliations:** 1 Department of Epidemiology, Johns Hopkins Bloomberg School of Public Health, Baltimore, MD 21205, USA; 2 Epicentre, Paris 75012, France; 3 Department of International Health, Johns Hopkins Bloomberg School of Public Health, Baltimore, MD 21205, USA; 4 Infectious Diseases Division, International Centre for Diarrhoeal Disease Research, Bangladesh (icddr,b), Dhaka 1212, Bangladesh; 5 PaxVax Inc., Redwood City, CA 94065, USA; 6 Division of Infectious Diseases, Massachusetts General Hospital, Boston, MA 02114, USA; 7 Department of Medicine, Harvard Medical School, Boston,MA 02115, USA; 8 Division of Global Health, Massachusetts General Hospital, Boston, MA 02114, USA; 9 Department of Pediatrics, Harvard Medical School, Boston, MA 02115, USA; 10 Department of Immunology and Infectious Diseases, Harvard T.H. Chan School of Public Health, Boston, MA 02115, USA; 11 Division of Infectious Diseases, University of Utah School of Medicine, Salt Lake City, UT 84132, USA; 12 Division of Microbiology and Immunology, University of Utah School of Medicine, Salt Lake City, UT 84132, USA

## Abstract

The development of new approaches to cholera control relies on an accurate understanding of cholera epidemiology. However, most information on cholera incidence lacks laboratory confirmation and instead relies on surveillance systems reporting medically attended acute watery diarrhea. If recent infections could be identified using serological markers, cross-sectional serosurveys would offer an alternative approach to measuring incidence. Here, we used 1569 serologic samples from a cohort of cholera cases and their uninfected contacts in Bangladesh to train machine learning models to identify recent *Vibrio cholerae* O1 infections. We found that an individual’s antibody profile contains information on the timing of *V. cholerae* O1 infections in the previous year. Our models using six serological markers accurately identified individuals in the Bangladesh cohort infected within the last year [cross-validated area under the curve (AUC), 93.4%; 95% confidence interval (CI), 92.1 to 94.7%], with a marginal performance decrease using models based on two markers (cross-validated AUC, 91.0%; 95% CI, 89.2 to 92.7%). We validated the performance of the two-marker model on data from a cohort of North American volunteers challenged with *V. cholerae* O1 (AUC range, 88.4 to 98.4%). In simulated serosurveys, our models accurately estimated annual incidence in both endemic and epidemic settings, even with sample sizes as small as 500 and annual incidence as low as two infections per 1000 individuals. Crosssectional serosurveys may be a viable approach to estimating cholera incidence.

## INTRODUCTION

Despite global efforts to improve access to safe water and adequate sanitation in many resource-poor settings, cholera remains a serious public health threat, killing more than 100,000 each year globally (*1*). Large epidemics in Africa, the Middle East, South Asia, and Haiti over the past decade have renewed global interest in the fight against cholera. In 2017, the World Health Organization (WHO)–backed Global Task Force for Cholera Control set a goal to end cholera as a public health threat by 2030 (*2*).

Accurate estimates of cholera incidence arekey for identifyingpriority intervention areas (i.e., cholera hotspots), evaluating new approaches to fighting cholera, and tracking/certifying progress in cholera control and elimination. Cholera incidence estimates are typically based on passive case-based reporting of acute watery diarrhea, and few cases are confirmed by culture or polymerase chain reaction. However, cholera symptom severity varies widely, access to health care varies, and case definitions can be insensitive or nonspecific. These factors lead to large uncertainties about *Vibrio cholerae* O1 infection incidence, its geographic distribution, and true disease burden.

Serosurveillance may provide one avenue to overcome existing cholera surveillance limitations and complement ongoing clinical surveillance efforts (*3*). However, serological correlates of recent *V. cholerae* O1 infection are not well established. Initial antibody responses are of the immunoglobulin M (IgM) isotype, which then progress to other isotypes such as IgG or IgA within days or weeks. Complement-fixing bactericidal antibodies directed at multiple *V. cholerae* antigens, known as vibriocidal antibodies, are the best-characterized immunologic marker of recent *V. cholerae* O1 infection. Vibriocidal antibody titers correlate with protection against cholera in household contacts of patients with cholera (*4*) and in human challenge studies (*5*) and have been used in numerous vaccine immunogenicity studies as the primary nonmechanistic correlate of protection (*6*). However, there is neither an established threshold at which protection is considered complete nor one for classifying someone as “recently infected.” In addition to vibriocidal antibodies, antibody responses to *V. cholerae* O1 serogroup antigens, including the O antigen of the lipopolysaccharide (LPS) and the B subunit of the cholera toxin (CTB), have been shown to rise and decline after infection, withmarked heterogeneity in kinetics between antibody isotypes (*7*). Flexible models capable of incorporating multiple cross-sectional serologic responses and demographic datamay improve our ability to accurately identify recent infections.

If individuals recently infected with *V. cholerae* O1 could be identified on the basis of their cross-sectional antibody profiles, this would provide an alternate measure of cholera incidence not subject to the biases of passive surveillance systems. The sharper picture of cholera epidemiology potentially provided by such a measure could play an instrumental role in enabling evidence-based approaches for targeting interventions, identifying the most effective cholera control tools, and tracking progress in fighting this ancient disease.

Here, we used data from a cohort of clinical cholera cases and their household contacts in Dhaka, Bangladesh and machine learning techniques to understand how different immunological markers of *V. cholerae* O1 infection can be used to identify recently infected individuals. We validated our approach using an independent set of serological data from a cohort of cholera-naïve North American volunteers challenged with *V. cholerae* O1, and we used simulation approaches to show how serological surveys can be used to reconstruct the size of epidemics.

## RESULTS

**Fig. 1:**
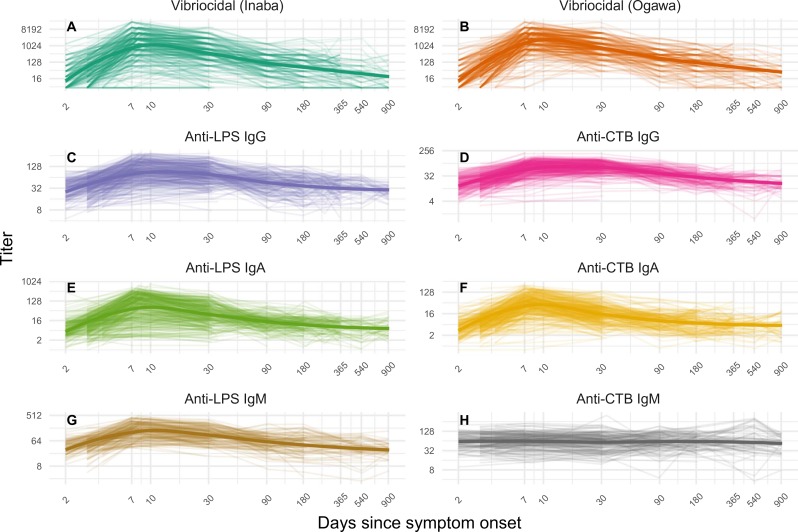
**Overviewof post-infection titer trajectories fromconfirmed cholera cases in Bangladesh cohort.** (**A** to **H**) Titer for a different antibody as a function of the number of days from (self-reported) symptom onset. The y axes are varied to aid visualization. Panels A and B show titers, whereas panels C to H are shown in ELISA units.

Weanalyzed data on 320 culture-confirmed cholera cases (287 *V. cholerae* O1 Ogawa and 33 *V. cholerae* O1 Inaba) enrolled at the International Centre for Diarrhoeal Disease Research, Bangladesh (icddr,b) cholera treatment center in Dhaka, Bangladesh between December 2006 and December 2015 (Table 1). Cases were followed for up to 915 days after symptom onset, with a median follow-up time of 91 days (Fig. 1 and fig. S1). Household contacts with no evidence of recent infection based on serial stool culture and serology in addition to symptom questionnaires (*n* = 58) were followed for up to 32 days (median, 30 days) and contributed additional data points for characterizing the background antibody distribution in the general population (Table 1, Fig. 1, and fig. S1).

**Table 1:** **Overview of participants in the Dhaka, Bangladesh cohort.** IQR, interquartile range.

	Cholera Cases	Household contacts
Number of participants	320	58
Median age of participants (IQR)	25 (8–35)	26 (18–34)
Male (%)	63.1	39.7
O blood group (%)	44.1	25.9
*V. cholerae* O1 Ogawa isolated (%)	89.7	-
Severely dehydrated at admission (%)	51.9	-

### Acute- and convalescent-phase antibody response kinetics suggest that infection signal in serum antibodies lasts at least 1 year after infection

**Fig. 2:**
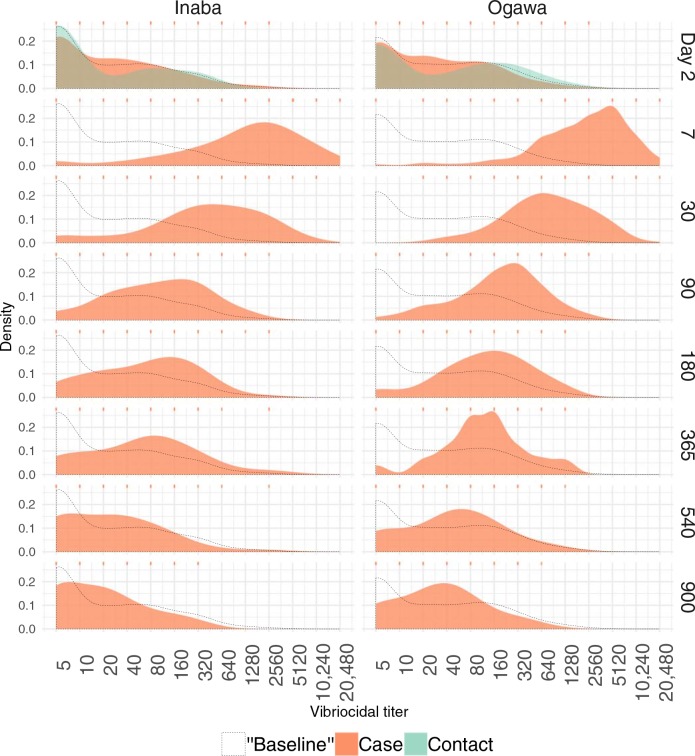
**Distribution of vibriocidal antibody titers by study visit day in the Bangladesh cohort.** Data from confirmed cholera cases are shown in orange and household contacts are shown in green. The dashed line represents the “baseline” titer distribution, a combined density of contacts across all visits and cases at first enrollment visit. Data are illustrated as ticks across *x* axes (top and bottom). Two-sided Kolmogorov-Smirnov tests to assess the similarity between distributions of titers at enrollment (day 2) for cases and contacts and found no significant differences for Ogawa (*P* = 0.4) and Inaba (*P* = 0.1).

At enrollment, about 2 days after symptom onset, cholera cases had similar titers to uninfected household contacts (Fig. 2 and figs. S2 and S3). Cases below 5 years of age had lower vibriocidal Inaba titers [1.22-fold decrease in median titer; 95% percentile bootstrap confidence interval (CI), 2.13 to 0.3-fold decrease], IgA titers against O1 LPS (0.68-fold decrease in median titer; 95% percentile bootstrap CI, 1.15 to 0.20-fold decrease), and higher IgG titers against CTB (0.75-fold increase in median titer; 95% percentile bootstrap CI, 0.39 to 1.12-fold increase) compared to those 5 years and older. We detected no significant differences in baseline titer by sex or blood group (e.g., O versus others; table S1). Whereas the distributions of antibodies against CTB and LPS at enrollment were unimodal in cases and contacts, the vibriocidal titer distributions in uninfected household contacts revealed two peaks. The first peak was close to the limit of detection of the assay, comprising 48.5% (95% CI, 35.3 to 61.4%) of the observations, suggesting that these groups comprised a mixture of individuals who were never or distally infected, and those more recently infected, whether symptomatic or asymptomatic (Fig. 2).

Between days 2 and 7 after symptom onset, the distribution of all titers, except anti-CTB IgM, increasedmarkedly in cases (Figs. 1 and 2 and figs. S2 and S3). Maximum titers for most cases were observed at day 7, althoughmore than a quarter of participants had theirmaximum IgG titers against LPS (25%) and CTB (36%) during the convalescent phase (i.e., on day 30). Vibriocidal antibodies, similar to antibodies against other pathogens (*8**,* *9*), had a biphasic decay, whereby they decreased log-linearly from the peak until 50 to 100 days after symptomonset and continued to decay at a slower log-linear rate through the end of follow-up (fig. S4). After 1 year, all responses except vibriocidal antibody titers returned to values close to thosemeasured during acute infection (1.1- to 1.4-fold higher at 1 year than baseline with all CIs spanning 1; figs. S4 to S6). The median vibriocidal titers 1 year after infection remained fourfold (Inaba: 95% percentile bootstrap CI, two- to eightfold) and eightfold (Ogawa: 95% percentile bootstrap CI, four- to eightfold) higher than during the acute phase of infection (Figs. 1 and 2), with 63% of cases having elevated vibriocidal (Ogawa) titers 1 year after infection.

### Vibriocidal antibody thresholds can identify recent infections

**Fig. 3:**
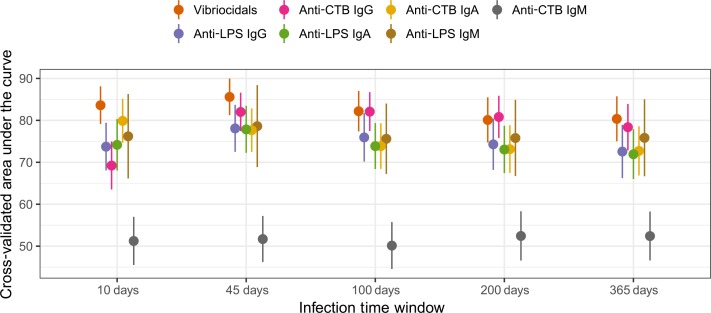
**cvAUC for each marker for different infection time windows.** Error bars represent the 95% CIs. Themarker labeled “Vibriocidals” represents using the maximum of each person’sOgawa and Inaba vibriocidal titers. Note that data on anti-LPS IgM (brown) and anti-CTB IgM (gray) were only available on a subset (*n* = 202) of participants.

We first explored the performance of single antibody and isotype thresholds in identifying individuals infected in the past 10, 45, 100, 200, or 365 days. Selecting thresholds that maximized combined sensitivity and specificity showed that vibriocidal antibodies outperformed all othermarkers or had similar performance to the best marker, at all times considered in cross-validation tests (Fig. 3 and tables S2 and S3). At 100- and 200-day infection windows, anti-CTB IgG antibodies had similar performance to vibriocidal antibodies (Fig. 3 and table S2). A vibriocidal antibody threshold of 320 (i.e., titer ≥ 320) correctly identified 80.6% (95% CI, 70.8 to 89.6%) of those infected in the last year, while limiting the false-positive rate to 17.0% (95% CI, 7.9 to 27.0%).

Across all thresholds, vibriocidal antibodies had an area under the cross-validated receiver operating characteristic curve (cvAUC), sometimes referred to as classifier accuracy, of 80.4% (95% CI, 75.0 to 85.7%) for identifying infections occurring in the last year. The nextbest performing marker was anti-CTB IgG antibodies (cvAUC, 78.4; 95% CI, 72.9 to 83.9%), followed by anti-LPS IgM (cvAUC, 75.8; 95% CI, 66.7 to 85.0%), anti-CTB IgA (cvAUC, 72.6; 95% CI, 66.2 to 78.9%), anti-LPS IgG (cvAUC, 76.6; 95% CI, 66.2 to 78.9%), and anti- LPS IgA (cvAUC, 71.9; 95% CI, 66.0 to 77.9%). Performance was similar across other infection windows (table S2). Although vibriocidal responses are thought to be largely IgM responses to the O-specific polysaccharide of the LPS antigen (*10*), anti-LPS IgM, which was only tested for in a subset of 202 participants (163 cases and 34 contacts), had poorer performance than vibriocidal titers (Fig. 3 and table S2). Single-antibody performance was similar between those below 5 years of age and those 5 years and older (fig. S7), except for the apparent increase in discriminatory power of anti-LPS IgA over longer time windows for ages below 5 years. However, the sample size of young children (below 5 years of age) was small (n = 24).

### Cross-sectional antibody profiles and machine learning methods improve classification of recent infections

**Fig. 4:**
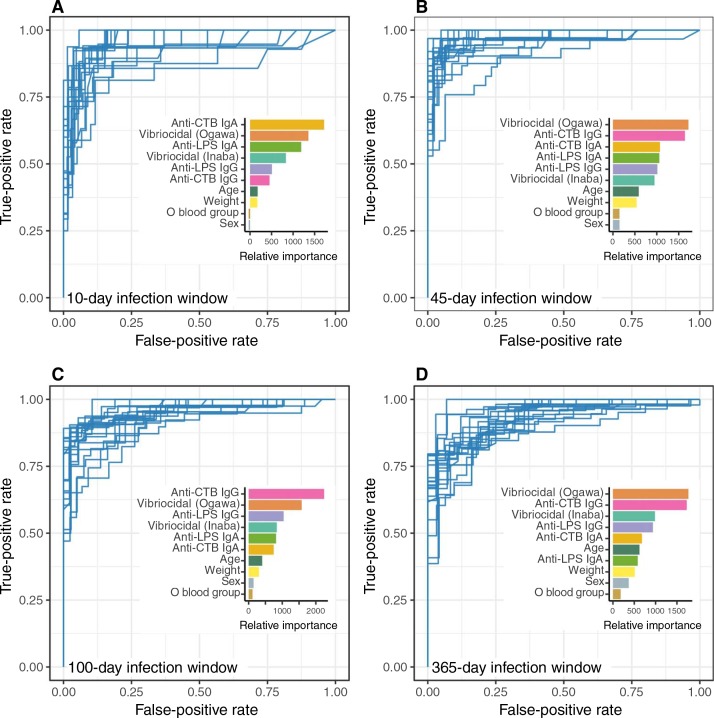
**cvAUCs and variable importance from random forest models by infection time window.** Blue curves represent individual cross-validated receiver operating characteristic curves from 20-fold cross validation of the random forest model over different infection time windows (**A** to **D**). Insets for each panel show the distribution of relative importance of each variable (median across cross-validation folds), with larger values representing parameters with more influence in the final model prediction as assessed through a permutation test procedure.

We used random forest models to understand whether information on multiple serologicalmarkers and demographic data could be combined to identify recent infectionsmore accurately than any singlemarker.We found that random forest models fit to individual cross-sectional antibody profiles and that demographics had higher sensitivity and specificity for identifying recent infections (i.e., those having occurred in the preceding 10, 45, 100, 200, or 365 days; Fig. 4 and fig. S8). These models performed well across infection time windows of up to 1 year, with cvAUCs ranging from 93.4 to 97.1% (Table 2 and table S4).

**Table 2:** **cvAUCs from random forest models fit to Bangladesh data by infection time window.** The full model included all markers and demographics (those shown in Fig. 4 panels). The two-marker model used only the top two markers from the full random forest model for each window, and the enzymelinked immunosorbent assay (ELISA)–only model used anti-CTB and anti-LPS IgA and IgG titers. Vibriocidal Ogawa titers were used in all two-marker models, the 10-day model used anti-CTB IgA, and the others used anti-CTB IgG. Estimates of performance for models fit to the subset of data that have IgM measurements are included in table S4. The 95% CIs are shown in parentheses.

Infection time window					
Model	10 days	45 days	100 days	200 days	365 days
Full model	94.5 (93.1–95.9)	97.1 (96.2–97.9)	95.0 (94.1–96.0)	93.6 (92.4–94.8)	93.4 (92.1–94.7)
Two markers	91.3 (89.0–93.7)	94.3 (92.9–95.7)	93.5 (92.2–94.8)	91.6 (90.1–93.1)	91.0 (89.2–92.7
ELISA only	90.1 (88.0–92.2.0)	93.6 (92.3–95.0)	91.9 (90.6–93.2)	89.8 (88.3–91.3)	87.0 (85.2–88.9)

In general, vibriocidal antibodies (Ogawa, as they were the most common in this cohort) and anti-CTB IgG were most important for the fit of the models; however, at the shortest infection window considered (10 days), anti-CTB IgA was similarly important (Fig. 4 and fig. S8). Demographic characteristics, including age and blood group, contributed little to the model fit across all time windows considered (Fig. 4, fig. S8, and table S5). Through the exploration of random forest models fit to data from cases only (no household contacts), we found that classifier performance in distinguishing infections that occurred in adjacent time windows diminished with time since infection (table S6). For example, we estimated a cvAUC of 66.6% (95% CI, 59.6 to 73.7%) for distinguishing those infected 100 to 200 days before compared to those infected 200 to 365 days before.

### Reducing data leads to small reductions in performance

Because analyzing serum samples for six to eight markers may not always be feasible, we explored the performance of simpler random forest models relying on the twomost influential antibodies identified for eachtime window (vibriocidalOgawa for all and anti-CTB IgG for all but the 10-day model; Table 2) and found that cvAUCs were similar to those using the entire antibody and demographic profile. These two-marker models have cvAUCs ranging from 91.0% (versus 93.4% for the full model) for a 1-year window to 94.3% (versus 97.1% for the full model) for a 45-day window (Table 2).

The vibriocidal assay is more time consuming and expensive than the ELISA methods used for the other potential biomarkers that we considered.Hence, we estimated the performance of models based only onfourELISA-basedassays (CTBandLPS IgAand IgG) andfound that the cvAUCs were reduced by less than 10%fromthe full model across all time windows (Table 2) and ranged from 87.0% (365-day window) to 93.6% (45-day window). ELISA-based models fit to the dataset with IgM available had slightly better performance than models without the inclusion of anti-CTB and anti-LPS IgM (table S4): The cvAUCs from these ELISA models with IgM isotypes ranged from 92.5% (versus 87.0% for 365-day window) to 94.8% (versus 93.6% for 45-day time window).

### Models perform well in external validation set of North American volunteers

Although our models were able to classify recent infections within a cohort in Bangladesh, where cholera is hyperendemic, it was unclear whether they would generalize to other settings, particularly to cholera epidemics in previously naïve populations. We used data from 38 healthyNorth American adult volunteers challengedwith *V. cholerae* O1 Inaba (*11*) who were then followed serologically for 6 months (figs. S9 to S11). Applying the vibriocidal thresholds derived from the Bangladesh cohort to the North American volunteers yielded equivalent or higher sensitivity and specificity as estimated for 45-, 100-, and 200-day infection windows in the Bangladesh dataset (table S7). Thresholds for CTB antibodies derived from the Bangladesh cohort, when considered alone, had low sensitivity for identifying recent infections (69.3 to 70.7% for IgG and 37.0 to 44.7% for IgA; table S7).

**Fig. 5:**
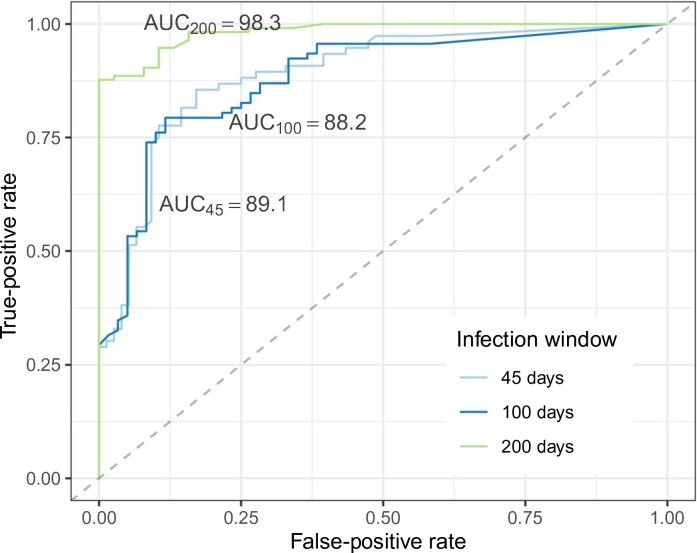
**Receiver operating characteristic curves for the external validation dataset of North American volunteers challenged with *V. cholerae* O1 Inaba.** Two-marker (vibriocidal and anti-CTB IgG) models were used for this because other antibody measures were not available in this cohort. Three curves are plotted, each using a different infection time window.

Two-marker random forest models, trained with data from Bangladesh, accurately identified recent infections inNorthAmerican volunteers after experimental infection. The AUC for a 200-day infection window was 98.2% (versus cvAUC of 91.6% in the Bangladesh data), with an AUC of 88.2% (versus 93.5% in Bangladesh data) for a 100-day window and 89.1% (versus 94.3% in Bangladesh data) for a 45-day window (Fig. 5). We did not test the full model, as only vibriocidal and CTB antibodies were available for this cohort. These results suggest that identification of recent infections (within 6 months) across populations with vastly different cholera epidemiology may be possible with this approach.

### Incidence can be estimated in simulated cross-sectional serological surveys

**Fig. 6:**
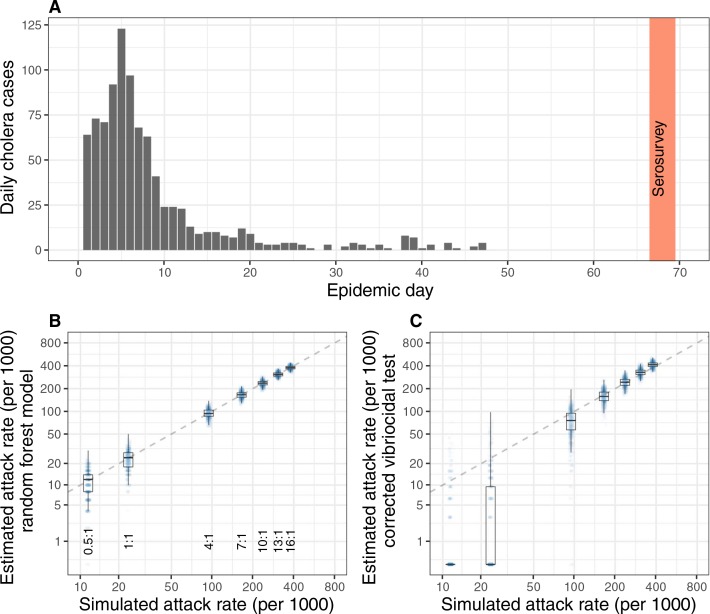
**Performance of random forest models and corrected vibriocidal test in estimating the infection attack rate in simulated post-epidemic serosurveys.** (**A**) Simulated epidemics had the same shape as that observed in an internally displaced person camp in South Sudan (*12*). The timing of the simulated serosurveys is shown as a vertical orange bar. (**B**) Infection attack rate estimates from the random forest model for different assumed case-to-infection ratios and a serosurvey sample size of 500 individuals. The dashed line represents the true simulated incidence, and numbers such as 0.5:1 represent the simulated infection-to-case ratio. For example, 4:1 represents simulations that followed an epidemiccurve with the same shape as that shown in (A) but with four times more infections than reported suspected cases. (C) Infection attack rate estimates from using a vibriocidal threshold of 320 but corrected for the estimated sensitivity and specificity over a 200-day infection window. The boxplots in (B) and (C) represent the median and IQR of the estimated attack rate, with the lines extending from each box representing 1.5 times the 25th or 75th percentile.

The performance of the cross-sectional antibody models suggests the possibility of reliably estimating recent infection incidence from serological surveys, which could provide a complementary approach to the use of clinical surveillance data. As a proof-of-concept, we simulated endemic and epidemic *V. cholerae* O1 transmission in synthetic populations. Each individual was assigned to demographic characteristics to mirror the Bangladesh cohort and, when infected, had an antibody profile drawn from smoothed antibody trajectories of members of that cohort. Endemic simulations assumed a constant hazard of infection, whereas epidemics were assumed to have an epidemic curve of the same shape as a 2014 outbreak in a displaced persons camp in South Sudan (Fig. 6A) (*12*), assuming varying ratios of medically attended cholera cases to infections.

In cholera-endemic populations, we found that random forest models could estimate the proportion of the population infected in the past year from small serosurveys (500 to 3000 individuals), although with a small positive bias (11.8 to 12.5% overestimation across simulations; table S8). Simulated serosurveys in endemic populations with an annual incidence rate of 5 per 1000 individuals had a mean of 5.6 per 1000 individuals [mean absolute error (MAE), 37.2%;mean bias, 12.0%]. Increasing sample size to 3000 decreased the MAE (22.6%). Using only (corrected) vibriocidal antibody thresholds led to larger MAE (20.4 to 438%) and bias in incidence estimates compared to the random forest model, especially when simulated annual incidence was low (table S8).

In epidemics similar to one observed in a South Sudan internally displaced persons camp [population, 39,000 (12)], we simulated serosurveys 3 weeks after the last case (~2 months after the peak of the epidemic curve) and found that the true epidemic size could be estimated with MAE ranging from 2.1 to 32.7% (Fig. 6). For epidemics with a simulated infection attack rate of 95 per 1000 individuals (infection-to-case ratio of 4:1), the MAE ranged from 4.3 to 11.0% with sample sizes from 500 to 3000. Incidence estimates based on vibriocidal antibody tests alone had larger error (MAE range, 5.2 to 99.7%), and incidence in simulations with low attack rates was highly underestimated (70% mean underestimation in serosurveyswith 500 individuals with an incidence rate of 23.6 per 1000 individuals).

## DISCUSSION

Here, we have shown that cross-sectional antibody profiles can be used to identify individuals recently infected with *V. cholerae* O1 with high sensitivity and specificity both in the hyperendemic Bangladeshi population used to train the model and in a separate population of cholera-naïve individuals from North America. We confirmed the importance of vibriocidal antibodies as amarker for recent infection and showed that the addition of other serologic markers provided greater resolution to reliably identify recent infections. These results provide a pathway for new approaches to assessing the burden of cholera across different populations, potentially overcoming many of the shortcomings of traditional clinic- and microbiology-based surveillance systems.

Measures of cholera incidence from serosurveys could help create more robust cholera surveillance systems, thereby improving our understanding of cholera epidemiology and providing new tools to track progress in the global fight against cholera. Clinical surveillance continues as an irreplaceable component of surveillance systems to identify microbiologic trends (e.g., antimicrobial resistance), trends in severity of medically attended disease, and concomitant enteric disease burden. The addition of routine serosurveys in “cholera hotspots” and after large epidemics can help us better understand the true trends in transmission, over time and across geographies (*13*), unaffected by unknown changes in care-seeking behavior and reporting system failures that often occur in cholera-affected areas. Serologically derived estimates of incidence may not need to be based on serum collected solely for cholera; instead, they could leverage existing nationally representative serosurveys including Demographic and Health Surveys (*14*) and the Population-based HIV Impact Assessment Surveys (*15*), conducted across many countries in sub-Saharan Africa.

Although our results indicate that we now have tools to make serologically derived estimates of recent incidence of cholera, more work can be done to improve their field readiness, cost, and generalizability. Universal protocols for the vibriocidal assays and ELISAs including standard controls such as monoclonal antibodies (*16*) may help ensure that numeric values from one laboratory are comparable across other laboratories. Development and validation of highthroughput vibriocidal methods and more field-adapted blood collection methods (*17*) will make serologic surveys for cholera more affordable and easier to implement in challenging environments. We highlighted the ability of our models to predict the proportion of the population recently infected in one relatively short epidemic; however, further analyses are needed to understand performance across a suite of epidemic shapes and durations to provide practical guidance for public health decision makers and researchers. In the case of longer epidemics or more endemic transmission, it may be that multiple serosurveyswill be needed to estimate the infection attack rate, although when to optimally conduct the surveys and howto combine their results remain an area of future research.

These analyses have a number of limitations. First, we used data from household contacts and the enrollment visit for cases to represent the “background” distribution of antibodies in the population. To reduce misclassification, we excluded contacts who reported diarrhea, had stool culture positive for *V. cholerae* O1, and/or had more than a twofold rise in vibriocidal titer during follow-up, although this was unlikely sufficient to fully eliminate misclassification as suggested by the bimodal vibriocidal distribution (*18*). Although the titers of cases at enrollment were similar to contacts, some cases may have already had a rise in antibodies at the time they enrolled in the study. This potential misclassification of both cases and contacts would lead us to underestimate the ability of our models to identify recent infections (table S9).

Although our models performed well across two epidemiologically distinct populations, the transportability of these models across settings with different mixes of historical exposure, proportion of symptomatic cases (sometimes linked to O blood group), and age distributions remains to be determined. The data that we used to train the models included only symptomatic *V. cholerae* O1 infections, although a high proportion of *V. cholerae* O1 infections are asymptomatic and differences in post-infection antibody kinetics by symptom severity are not well understood. The probability of becoming symptomatic may be linked to previous exposure to the bacteria and the inoculum size (*19*), both of which have the potential to modify the immune response to infection. Studying the kinetics of asymptomatic infections requires a unique setting, such as households in low-incidence areas, where the date of probable exposure can be identified (or bounded). Our validation set of North American volunteers challenged with *V. cholerae* contained asymptomatic (5%) and mildly symptomatic (39%) patient and those with moderate-to-severe disease (56%). The proportion of serologic profiles correctly classified did not vary by severity, hinting that model performance may be insensitive to symptom severity. Similarly, although our models did not perform better by including age, we had few children below 5 years of age in the study (*n* = 24) and antibody kinetics may vary within this population as suggested by our decay models.

We found that antibodies to the cholera toxin (IgG and IgA) were important for classifying recent infection in Bangladesh. However, they alonewerenotpredictive intheNorth American volunteers,which may be related to differences in previous exposure to the heat-labile enterotoxin of enterotoxigenic *Escherichia coli*, which is highly homologous with cholera toxin. Antibodies of IgM isotypes were not among the most influential predictors of recent infection in the Bangladesh cohort. However, anti-LPS IgMmay bemore important among primary infections, and future models tailored to settings with minimal historical exposure may consider IgM isotypes more heavily. Our random forest model performed well with training and validation datasets (external and internal). However, as new data become available, they may warrant the updating of a new single model or the development of new models for different epidemiologic settings or populations.

In our simulated serosurveys, we generated antibody profiles for individuals from smoothed versions of the antibody trajectories of participants used to fit the prediction models, which may lead to overly optimistic results. As the time from infection increases, the model’s ability to discriminate between two individuals infected close together in time diminishes. Therefore, when using serology to estimate the final size of an outbreak or incidence in the previous year, future work may take into account the likely distribution of infection times over the period of interest, potentially using clinic-based epidemic curves as Bayesian priors. This time-varying performance of themodels may be responsible for small positive bias in incidence estimates in modeled endemic settings. As killed whole-cell oral cholera vaccines become more broadly used in cholera control, populations will increasingly comprise a mix of individuals exposed only to the vaccine, those exposed only to the live bacteria, and those exposed to both. Our current models are unable to classify these different individuals without additional data on the antibody kinetics after vaccination (including breakthrough infections in vaccinated individuals), the inclusion of additional biomarkers (e.g., antibodies to the O139 antigen that is included in bivalent vaccines but rarely circulates), the time since potential vaccine exposure (little to no vaccination is often available in between discrete vaccination campaigns), and new statistical methods.

Because of nonspecific clinical symptoms of cholera and the concentration of its occurrence in areas with limited laboratory and surveillance resources, measuring the true incidence has long been a challenge. Using a deeper understanding of *V. cholerae* O1 postinfection antibody dynamics, the data and methods presented here may open up new ways of measuring cholera burden in endemic and epidemic settings based on serosurveys. In doing so, it may help lead to fundamental improvements in our understanding of cholera epidemiology needed to effectively achieve the goal of ending cholera as a public health threat by 2030.

## MATERIALS AND METHODS

### Study design

Our research objective was to explore the potential for using crosssectional serological antibody measurements to identify recent *V. cholerae* O1 infections.We primarily relied on two datasets from previously published studies to train and test models: one from Dhaka, Bangladesh (*20*, *21*) and an external validation dataset from North American volunteers (*11*).

The primary data used to characterize the post-infection immune response kinetics and to train our models composed of a cohort of confirmed cholera cases seeking care at the icddr,b Dhaka hospital (Bangladesh) and their household contacts from previously described studies (*20*, *21*). Patients 2 to 60 years old presenting to the icddr,b Dhaka hospital between 2006 and 2015 with severe acute watery diarrhea, a stool sample positive for *V. cholerae* O1, and no other identified diarrheal pathogen with no other serious comorbidities who resided in and around Dhakawere eligible to participate in the study. Study staff approached a convenience sample of eligible patients receiving care and asked for consent to participate in the study. After obtaining written informed consent, study staff attempted to collect a venous blood sample on the second day of hospitalization and on or around days 7, 30, 90, 180, 270, 365, 540, 720, and 900 after symptom onset. The exact days from the onset of diarrhea or vomiting are used in analyses, but for simplicity in descriptive analyses and narrative, we refer to these days as “day 7,” “day 30,” etc. (fig. S1).

Household contacts were defined as individuals who shared a cooking pot with the index case for three or more consecutive days preceding the cholera episode. After consent, blood samples were collected from contacts on the day of enrollment (day 2) and on days 7 and 30 after hospitalization of the index case with rectal swabs collected daily during the first 10 days after enrollment. These individuals were used as controls in analyses to provide more data on the background antibody distribution in addition to day 0 measurements of confirmed cases.

To minimize the risk of misclassification, we excluded person visits starting from any visit where individuals had more than a twofold rise in vibriocidal titer from his/her previous visit after the observed convalescent peak (in cases only). We excluded data from household contacts who had a vibriocidal titer greater than 640 at all study visits because this was suggestive of recent infection. Because of the high risk of secondary cases within households (*18*), we excluded household contact visits after they reported having acute watery diarrhea or when we detected a stool culture positive for *V. cholerae* O1 or O139. After applying these exclusion criteria, this “uninfected” group of contacts included potentially both uninfected and recent but asymptomatic infections.

To assess the external validity of our models, we used data from a cohort of 38 NorthAmerican volunteers challengedwith cholera aspart of the placebo armof a vaccine clinical trial (*11*). These adult volunteers ranged in age from 19 to 44 years (median age, 30.5 years) and had no known history of cholera. They were challenged with virulent *V. cholera* O1 El Tor Inaba N16961 and had blood collected on days 0, 10, 28, 90, and 170 after challenge. Vibriocidal antibodies and anti-CTB antibodies (IgA, IgM, and IgG) were assessed using the same methods as the samples from Bangladesh.

### Laboratory analyses

Plasma was separated from venous blood samples from each visit and stored at −80°C. We measured vibriocidal antibody titers in plasma by standard technique as previously described using guinea pig complement and *V. cholerae* O1 Ogawa (X-25049) or Inaba (T-19479) as the target organism for the Bangladesh samples and *V. cholerae* O1 Ogawa (PIC158) or Inaba (PIC018) as the target organism for the North American samples (4, 22). Plasma was serially diluted in a 96-well plate, in duplicate (on the same plate), until a final dilution of 1:10,240. Bacteria and complement were added to each well before incubation on an orbital shaker at 37°C for 1 hour. Last, 150 ml of brain heart infusion media was added to each well, and the plate was incubated for 2 to 4 hours at 37°C until the positive growth control (bacteria only) reached an optical density at 600 nm (OD_600_) of 0.20 to 0.28. Pooled serum from acute- and convalescent-confirmed cholera cases was used as a positive control. Experiments were rejected and repeated if the titer of the positive control was more than one dilution from the “known” value. We defined the vibriocidal titer as the reciprocal of the highest dilution resulting in 50% reduction of the OD compared to that of the average of the three positive controlwells without plasma. We measured plasma isotype-specific antibody responses against V. cholerae O1 LPS (serotype matched to index case serotype) and CTB using standardized ELISA methods as previously described (22). We read plates kinetically at 450 nm for 5 min and normalized themaximum rate of change inODin milliabsorbance units perminute across plates by calculating the ratio of the test sample to a standard of pooled convalescent-phase sera from patients previously infected with cholera included on each plate. ABO blood group type was determined through a slide agglutination test (Biotec Laboratories) according to the manufacturer’s instructions.

The objective of the main analysis was to identify individuals recently infected with *V. cholerae* O1. Although there is no agreed upon definition of “recent,” we focused on five time frames (5 to 10 days, 5 to 45 days, 5 to 100 days, 5 to 200 days, and 5 to 365 days) that may be of interest for epidemiologic and public health decision-making. We excluded the acute and early convalescent periods (roughly the first 5 days) because there was a significant heterogeneity between individuals, and the titer values on the rising portion of the titer trajectory were very hard to distinguish from their counterparts on the declining side of the trajectory. We referred to each time window by the upper limit (e.g., “100-day window” refers to the 5-to 100-day window) for simplicity.

### Single-marker thresholds

First, we explored the performance of different single-marker thresholds for identifying individuals infected over different time frames. We assessed performance by estimating the 20-fold cvAUC, dividing individuals, rather than individual observations, into 1 of the 20 different folds. Inference on cvAUCs, which accounted for repeated observations per person, was performed using influence curvemethods as implemented in the cvAUC package in R (23).

We also explored the exact titer thresholds that led to maximization of the sensitivity and specificity combined [Youden index (24)]. First, for each exposure time window and antibody, we identified the antibody titer threshold that maximized the sensitivity and specificity of most of the 1000 training sets (made up of 70% of participants), with a single observation (time point) selected for each person. We then calculated the mean, median, and other quantiles of the distribution of sensitivity and specificity of this titer applied to the corresponding validation set.

### Random forest classification models

We used random forest models to identify recently infected individuals based on cross-sectional antibody titers and, in some models, blood type and demographic data. We built a series of models with a binary outcome of recent infection, considering different time windows (as described above). In each model, those infected inside the time window were considered “recently infected” and those cases infected outside the time window (or never), including “uninfected controls,” were considered “not recently infected.” We then fit the models using the randomForest R package with 1000 trees. We assessed model performance by estimating the 20-fold cvAUC. We estimated the 95% CIs for the AUCs with influence curves accounting for within-person correlation with cvAUC (23). We assessed variable importance within these different models using a permutation test–basedmetric,mean decrease in accuracy (25).

Given the correlated nature of our data within individuals, we also explored estimating AUCs using a subsampling cross-validation approach. We sampled a single observation per individual and then divided the observations between a training set (70%) used to fit the model and a testing set (30%) used to estimate the AUC. Repeating this procedure 500 times, we found that AUC estimates were almost identical to those found using full data from each individual and that the 2.5th and 97.5th percentiles of theAUCdistributions were similar to the estimated 95% CIs of cvAUCs.

### Simulated serosurveys and estimating cumulative incidence

To illustrate the utility of ourmodels applied to cross-sectional serological data, we modeled two settings, a cholera-endemic setting and a cholera-epidemic setting, each with 25,000 individuals. In the endemic setting, we assumed a constant hazard of infection with annual incidence rates of 2, 5, 10, 50, and 100 per 1000 population. For each incidence rate, we simulated 1000 independent populations in endemic transmission settings, sampled 500 to 3000 individuals, and classified each profile as being infected or not using both the randomforest model (full 1-year model with antibodies and demographics fit to the original data from Bangladesh) and the vibriocidal test. In each synthetic population, we chose the number of cases infected in the last year by multiplying the population size with the annual incidence rate. Assuming a constant incidence rate, 1.5 times this number were infected 1 to 2.5 years ago. The rest of the population was assumed to have been infected either never ormore than 2.5 years before and was assigned an infection time of infinity.Wethen estimated the mean annual incidence estimated across simulations, the MAE, and the mean bias. Estimates derived from vibriocidal antibodies alone were corrected for their sensitivity and specificity, where the corrected number of positive individuals (*n*_pos_) is a function of the sensitivity (sens) and specificity (spec) of the test, the number tested (n), and the number tested positive ($n_{\text{pos}}^{*}$)

$$n_{\text{pos}}=\max\left(\frac{n_{\text{pos}}^{*}+n\times \left(spec-1\right)}{sens+spec-1},0\right)$$

To explore more realistic simulated epidemics, we used the observed epidemic curve from an outbreak in a displaced person camp in South Sudan (*12*) and assumed that the observed cases represented a constant proportion of *V. cholerae* O1 infections through time, with infection-to-case ratios varying from 0.5:1 to 16:1. We simulated 500 epidemics for each infection-to-case ratio, estimated the proportion of the population exposed in the last 365 days based on randomly sampling 500, 1000, or 2500 individuals in 100 simulated serosurveys, and then applied the random forest model or vibriocidal threshold test (corrected).

We created individual antibody profiles for infected individuals in the simulations by linearly interpolating individual case’s longitudinal antibody trajectories from the Bangladesh cohort. To choose an antibody trajectory for a person infected t days ago, we randomly chose an antibody trajectory with follow-up time (≥t; uniformly across all individuals with data at that time point or greater) and used the estimated titers for each antibody at that time point. We explored the use of smoothed antibody trajectories using penalized splines in sensitivity analyses. For noninfected individuals or those infected more than 2.5 years before, we randomly chose antibody profiles from all followup visits of household contacts with no evidence of exposure or from profiles of cases at enrollment. All analyses were conducted with the R statistical computing language (version 3.2.3).

### Statistical analysis

We explored the distributions of antibody titers at different times using Gaussian kernel density estimation methods using the ggplot2 package in R (*26*). We compared differences in baseline titer for the different antibodies by sex, age group, and blood group using Mann-Whitney tests and applied a Bonferroni correction to account for the multiple tests being conducted (by antibody). To better characterize the bimodal distribution of contact titers, we constructed a two-component Gaussian mixture model of the log of the vibriocidal titers (maximum of Inaba and Ogawa) with JAGS (version 4.3.0_2) and implemented in R with runjags. Observations were treated as interval censored with the two Gaussian distributions assumed to share a common variance.We assigned a Gamma(0.01,0.01) prior distribution to the precision and a Dirichlet(1) to the mixing proportion parameter. We ran four chains of 75,000 iterations and used visual inspection and the Gelman-Rubin (R hat) statistic to assess convergence.

We modeled the post-peak decay of each antibody using a generalized additive mixed modeling framework, allowing for the relationship between days since symptomonset and titer to bemodeled as a monotonically decreasing P-spline using the scam package in R (*27*). Models included Gaussian random intercepts per person, and we explored variants that allowed for separate splines for participants below 5 years old and for those 5 years and older.Model fits were compared using deviance information criterion.

## SUPPLEMENTARY MATERIALS

www.sciencetranslationalmedicine.org/cgi/content/full/11/480/eaau6242/DC1

Fig. S1. Illustration of study visits by days since the symptom onset of the primary household case for cases and household contacts.

Fig. S2. Distribution of anti-CTB IgG, IgM, and IgA titers by study visit day for confirmed cholera cases (orange) and household contacts (light green) in the Bangladesh cohort.

Fig. S3. Distribution of anti-LPS IgG, IgM, and IgA titers by study visit day for confirmed cholera cases (orange) and household contacts (light green) in the Bangladesh cohort.

Fig. S4. Estimated vibriocidal decay curves for (gray) all cases, cases below 5 years old (blue), and cases 5 years and older (red).

Fig. S5. Estimated anti-CTB IgG and IgA titer decay curves for (gray) all cases, cases below 5 years old (blue), and cases 5 years and older (red).

Fig. S6. Estimated anti-LPS IgG, IgA, and IgM titer decay curves for (gray) all cases, cases below 5 years old (blue), and cases 5 years and older (red).

Fig. S7. cvAUC for each marker for different infection time windows by age group.

Fig. S8. cvAUC and variable importance from random forest models fit to subset of Bangladesh data with IgM measurement by infection time window.

Fig. S9. Distribution of baseline vibriocidal titers in North American volunteers (United States, orange) and household contacts in Bangladesh (Dhaka, green).

Fig. S10. Distribution of baseline anti-CTB IgG, IgM, and IgA titers in North American volunteers (United States, orange) and household contacts in Bangladesh (Dhaka, green).

Fig. S11. Distribution of anti-CTM IgG (red), IgM (green), and IgA (blue) titers by day after experimental infection among North American volunteers.

Table S1. Differences in (log_2_) median titer at baseline between subgroups for each marker.

Table S2. cvAUC for single markers within the Bangladesh cohort over different infection time windows.

Table S3. Thresholds (modal titer) and cross-validated sensitivity and specificity for single-antibody threshold tests within the Bangladesh cohort by infection time window.

Table S4. cvAUC from random forest models fit to IgM subset of Bangladesh data by infection time window.

Table S5. cvAUC from random forest models fit to Bangladesh data subset of blood type O negatives only.

Table S6. cvAUC from random forest models discriminating between infections occurring in different time windows.

Table S7. Sensitivity and specificity of single-antibody thresholds in an external validation set of North American volunteers (*n* = 38) over different time windows.

Table S8. Estimated mean annual incidence, MAE, and mean bias error from both random forest models and corrected vibriocidal tests.

Table S9. cvAUC for random forest models trained on a subset of data (*n* = 347) excluding potentially infected household contacts.

## Supplementary Material

Estimating cholera incidence with cross-sectional serology
