# Supplementary material for: Estimating cholera incidence with cross-sectional serology
Source: Sci Transl Med. 2019 Feb 20;11(480):eaau6242. doi: 10.1126/scitranslmed.aau6242 (PMC6430585; doi:10.1126/scitranslmed.aau6242)
Supplement: Estimating cholera incidence with cross-sectional serology [file STM-11-eaau6242-s001.pdf]

Science Translational Medicine
AAAS

www.sciencetranslationalmedicine.org/cgi/content/full/11/480/eaau6242/DC1

Supplementary Materials for

Estimating cholera incidence with cross-sectional serology

Andrew S. Azman*, Justin Lessler, Francisco J. Luquero, Taufiqur Rahman Bhuiyan, Ashraful Islam Khan, Fahima Chowdhury, Alamgir Kabir, Marc Gurwith, Ana A. Weil, Jason B. Harris, Stephen B. Calderwood, Edward T. Ryan, Firdausi Qadri, Daniel T. Leung

*Corresponding author. Email: azman@jhu.edu

Published 20 February 2019, *Sci. Transl. Med.* **11**, eaau6242 (2019)
DOI: 10.1126/scitranslmed.aau6242

This PDF file includes:

Fig. S1. Illustration of study visits by days since the symptom onset of the primary household case for cases and household contacts.
Fig. S2. Distribution of anti-CTB IgG, IgM, and IgA titers by study visit day for confirmed cholera cases (orange) and household contacts (light green) in the Bangladesh cohort.
Fig. S3. Distribution of anti-LPS IgG, IgM, and IgA titers by study visit day for confirmed cholera cases (orange) and household contacts (light green) in the Bangladesh cohort.
Fig. S4. Estimated vibriocidal decay curves for (gray) all cases, cases below 5 years old (blue), and cases 5 years and older (red).
Fig. S5. Estimated anti-CTB IgG and IgA titer decay curves for (gray) all cases, cases below 5 years old (blue), and cases 5 years and older (red).
Fig. S6. Estimated anti-LPS IgG, IgA, and IgM titer decay curves for (gray) all cases, cases below 5 years old (blue), and cases 5 years and older (red).
Fig. S7. cvAUC for each marker for different infection time windows by age group.
Fig. S8. cvAUC and variable importance from random forest models fit to subset of Bangladesh data with IgM measurement by infection time window.
Fig. S9. Distribution of baseline vibriocidal titers in North American volunteers (United States, orange) and household contacts in Bangladesh (Dhaka, green).
Fig. S10. Distribution of baseline anti-CTB IgG, IgM, and IgA titers in North American volunteers (United States, orange) and household contacts in Bangladesh (Dhaka, green).
Fig. S11. Distribution of anti-CTM IgG (red), IgM (green), and IgA (blue) titers by day after experimental infection among North American volunteers.
Table S1. Differences in ($\log_2$) median titer at baseline between subgroups for each marker.
Table S2. cvAUC for single markers within the Bangladesh cohort over different infection time windows.
Table S3. Thresholds (modal titer) and cross-validated sensitivity and specificity for single-antibody threshold tests within the Bangladesh cohort by infection time window.

Table S4. cvAUC from random forest models fit to IgM subset of Bangladesh data by infection time window.
Table S5. cvAUC from random forest models fit to Bangladesh data subset of blood type O negatives only.
Table S6. cvAUC from random forest models discriminating between infections occurring in different time windows.
Table S7. Sensitivity and specificity of single-antibody thresholds in an external validation set of North American volunteers (n = 38) over different time windows.
Table S8. Estimated mean annual incidence, MAE, and mean bias error from both random forest models and corrected vibriocidal tests.
Table S9. cvAUC for random forest models trained on a subset of data (n = 347) excluding potentially infected household contacts.

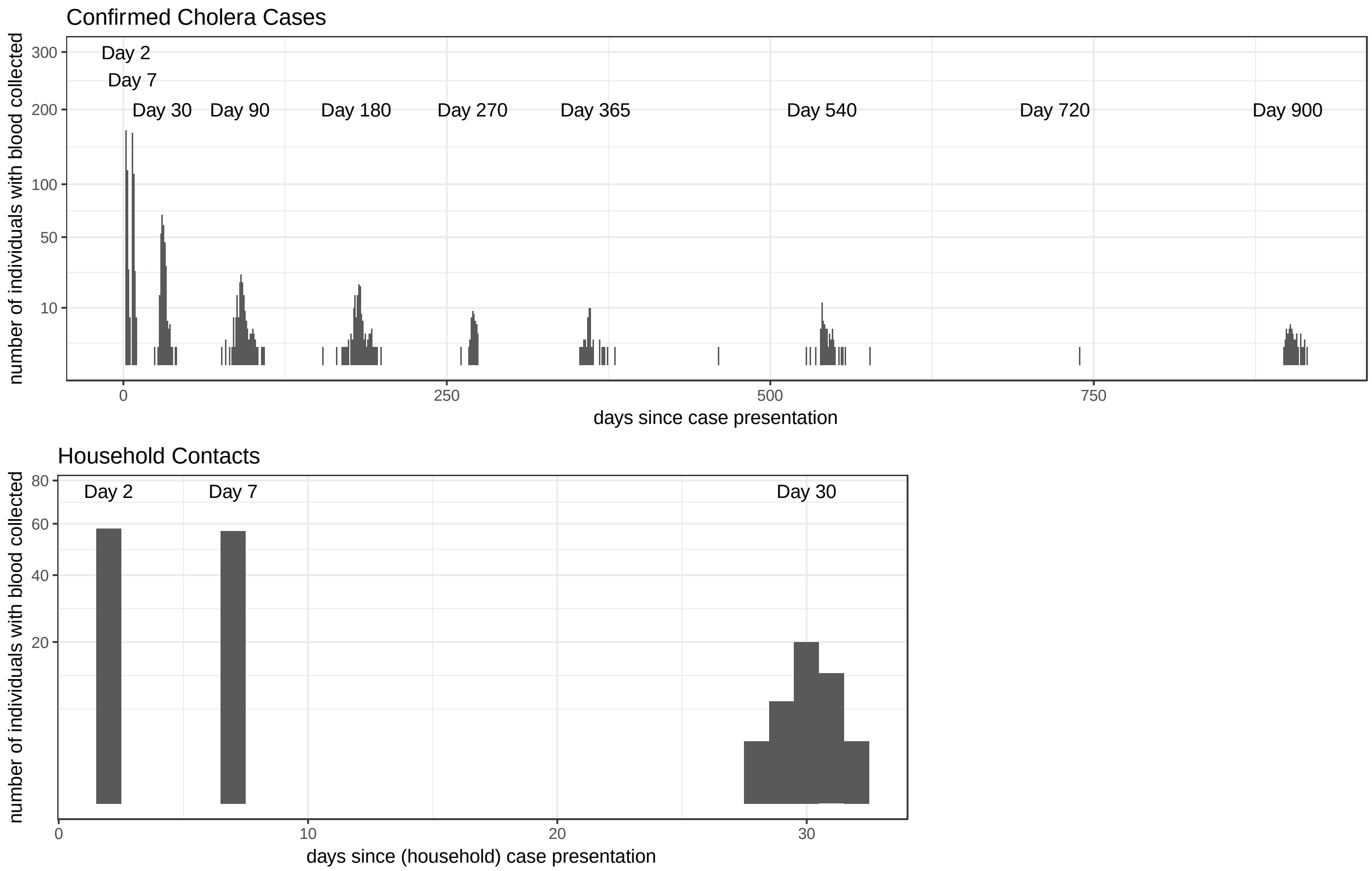

Fig. S1. Illustration of study visits by days since the symptom onset of the primary household case for cases and household contacts. Annotated text above each mode highlights the study visit grouping used in descriptive analyses. For example, all study visits around the mode marked 'Day 90' are considered day 90 in descriptive analyses in manuscript. However, the exact days since symptom onset are used in all models. Note that y-axes are shown with a square root transformation to help visualization.

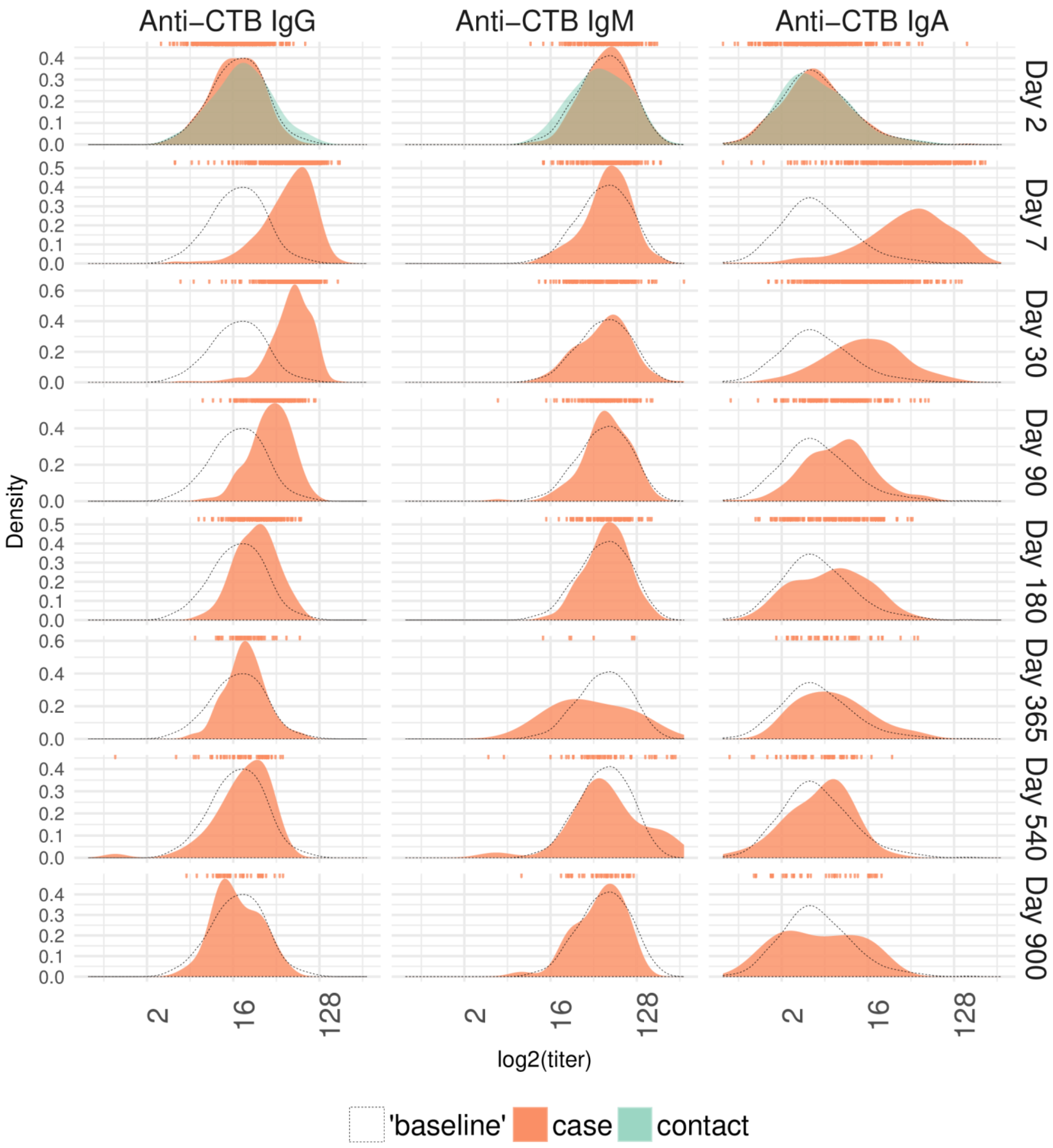

Fig. S2. Distribution of anti-CTB IgG, IgM, and IgA titers by study visit day for confirmed cholera cases (orange) and household contacts (light green) in the Bangladesh cohort. The dashed line represents the ‘baseline’ titer distribution, a combined density of contacts across all visits and cases at first enrollment visit. Data are illustrated as ticks across the top of the x-axes. We performed two-sided Kolmogorov-Smirnov tests to assess the similarity between distributions of titers at enrollment (day 2) for cases and contacts and found no significant differences for IgG (p=0.5), IgM (p=0.1) and IgA (p=0.3).

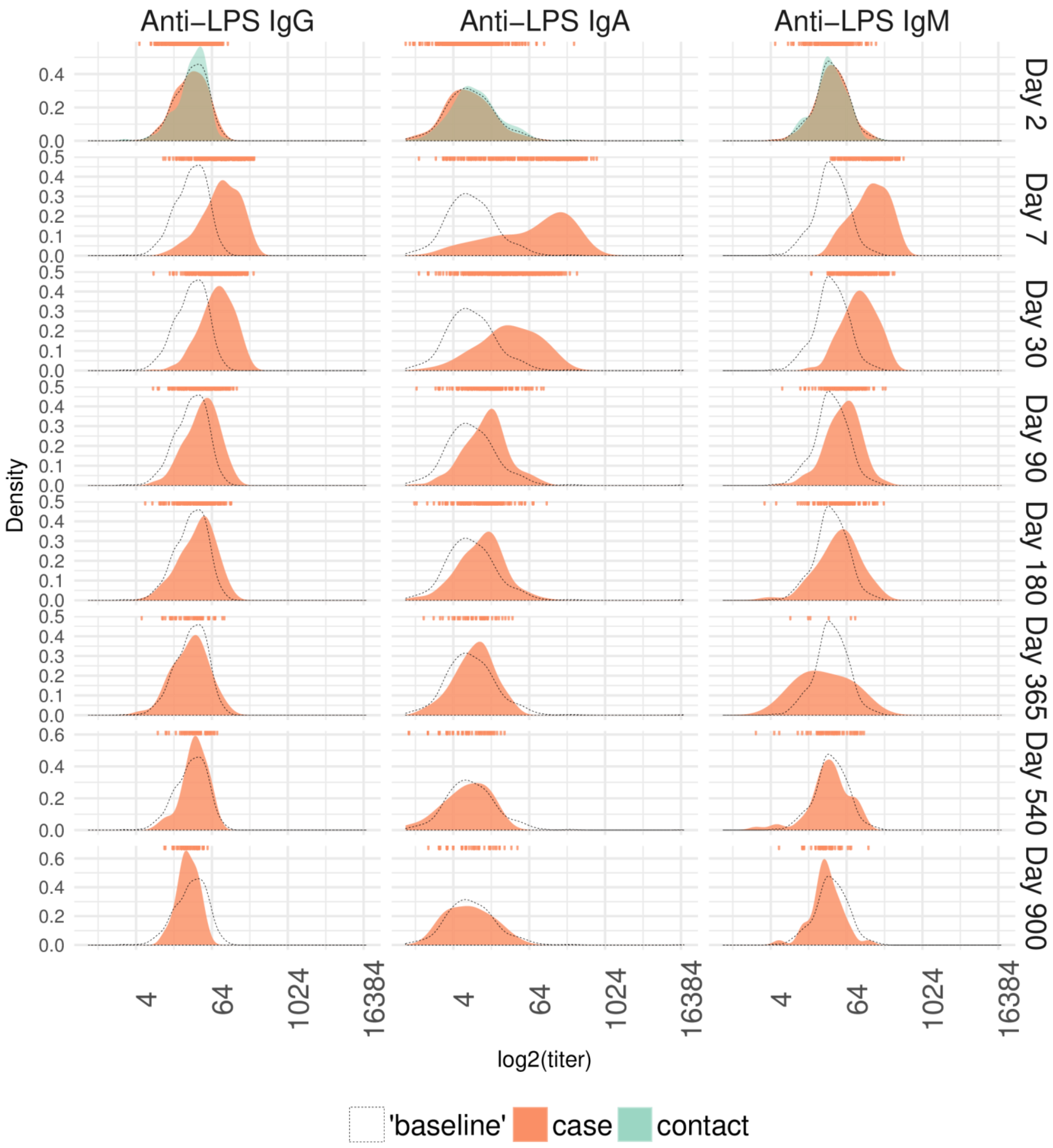

Fig. S3. Distribution of anti-LPS IgG, IgM, and IgA titers by study visit day for confirmed cholera cases (orange) and household contacts (light green) in the Bangladesh cohort. The dashed line represents the ‘baseline’ titer distribution, a combined density of contacts across all visits and cases at first enrollment visit. Data are illustrated as ticks across the top of the x-axes. We performed two-sided Kolmogorov-Smirnov tests to assess the similarity between distributions of titers at enrollment (day 2) for cases and contacts and found no significant differences for IgG (p=0.2), IgM (p=0.9) and IgA (p=0.1). Rectangles represent the interquartile range of titers at enrollment for each group for comparison purposes.

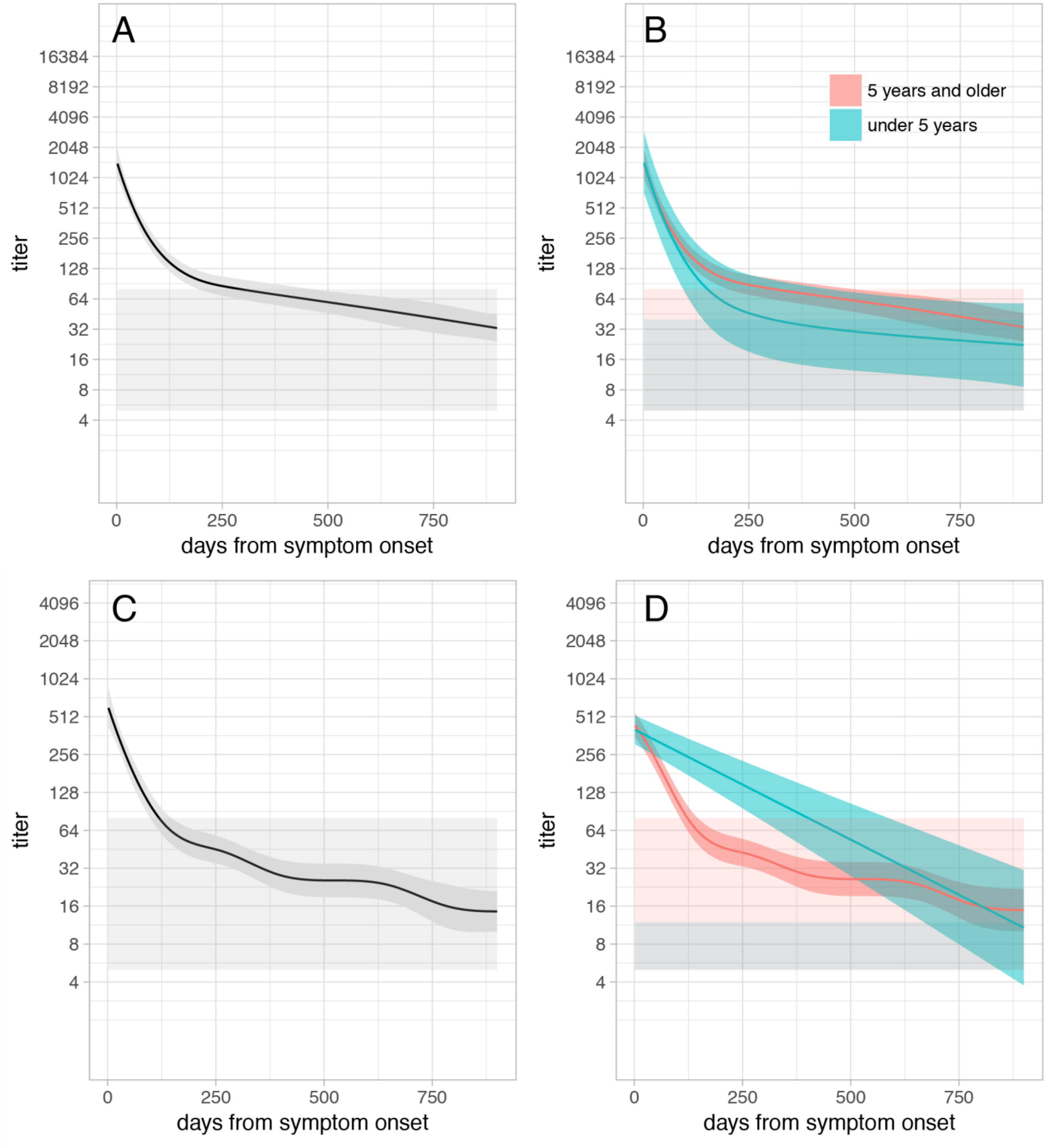

Fig. S4. Estimated vibriocidal decay curves for (gray) all cases, cases below 5 years old (blue), and cases 5 years and older (red). Estimates of vibriocidal Ogawa (A-B) and Inaba (C-D) decay curves are from a generalized mixed effects additive model with constrained (monotonically decreasing) P-splines for the titer decay. Solid lines represent mean estimates of the fixed effects with 95% confidence intervals shown in the surrounding envelopes. Rectangles represent the interquartile range of titers at enrollment for each group for comparison purposes.

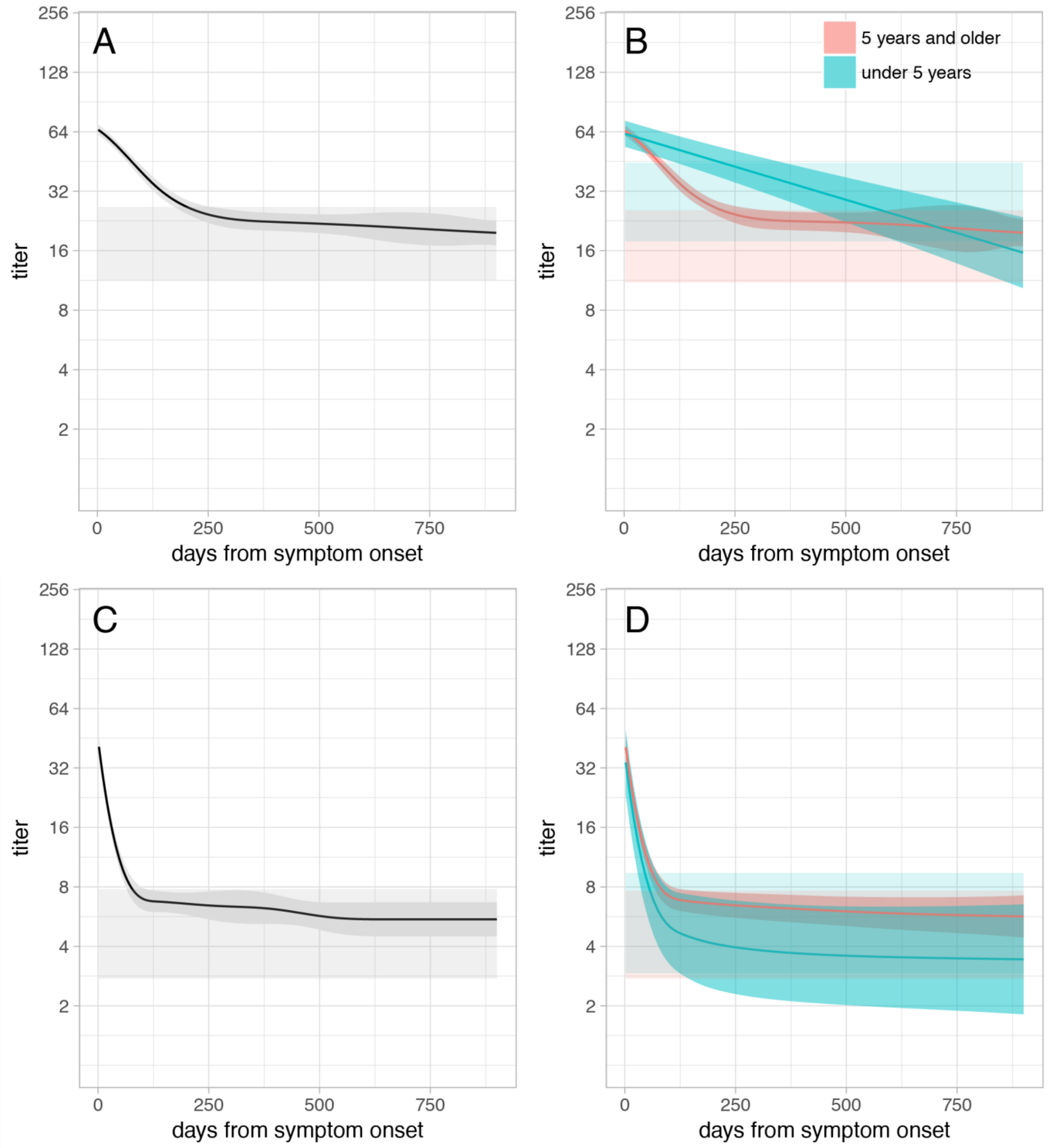

Fig. S5. Estimated anti-CTB IgG and IgA titer decay curves for (gray) all cases, cases below 5 years old (blue), and cases 5 years and older (red). Estimates of anti-CTB IgG (A-B) and anti-CTB IgA (C-D) decay curves are from a generalized mixed effects additive model with constrained (monotonically decreasing) P-splines for the titer decay. Solid lines represent mean estimates of the fixed effects with the 95% confidence intervals shown in the surrounding envelopes. Rectangles represent the interquartile range of titers at enrollment for each group for comparison purposes.

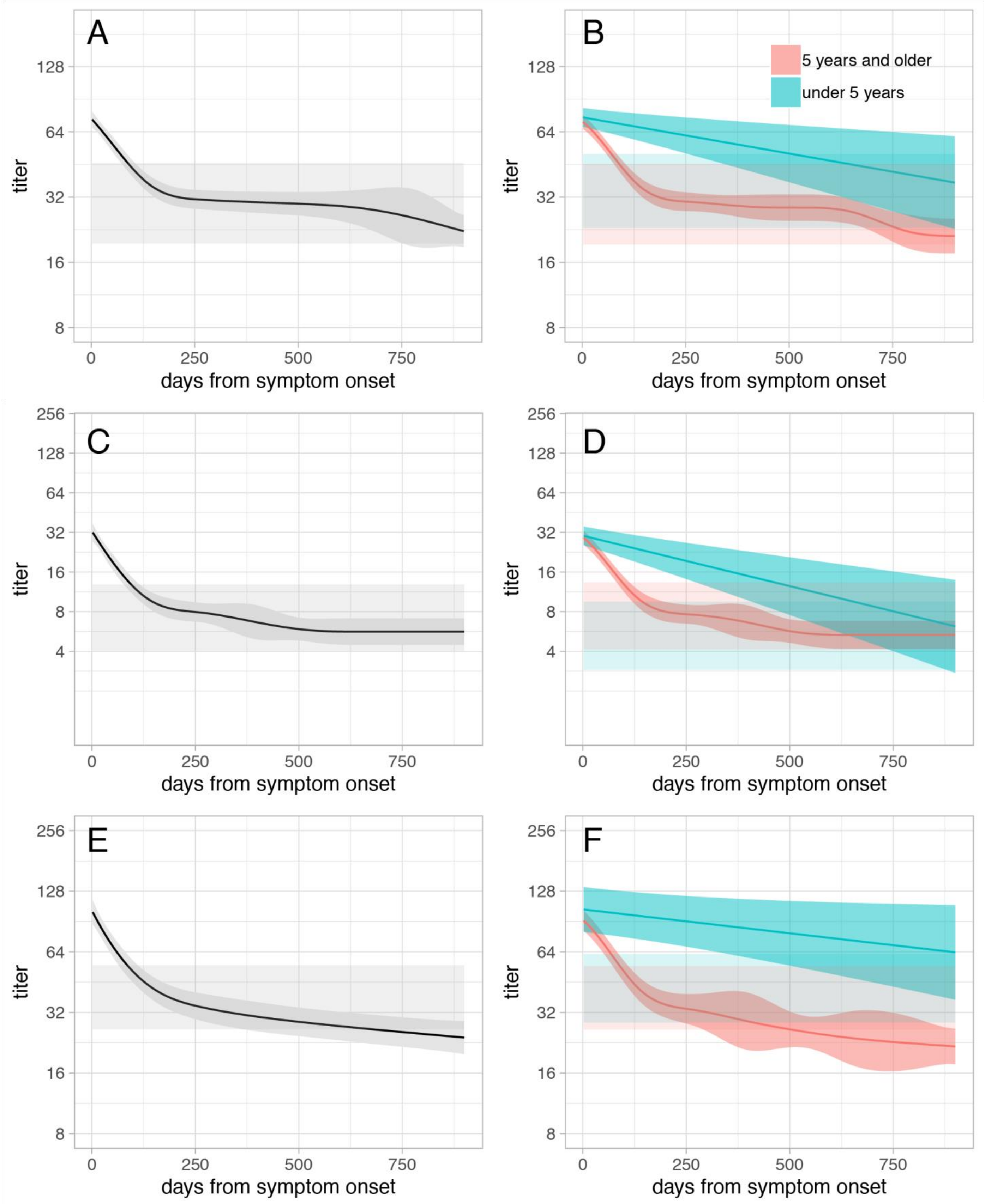

Fig. S6. Estimated anti-LPS IgG, IgA, and IgM titer decay curves for (gray) all cases, cases below 5 years old (blue), and cases 5 years and older (red). Estimates of anti-LPS IgG (A-B), IgA (C-D) and IgM (E-F) are from a generalized mixed effects additive model with constrained (monotonically decreasing) P-splines for the titer decay. Solid lines represent mean estimates of the fixed effects with the 95%

confidence intervals shown in the surrounding envelopes. Rectangles represent the interquartile range of titers at enrollment for each group for comparison purposes.

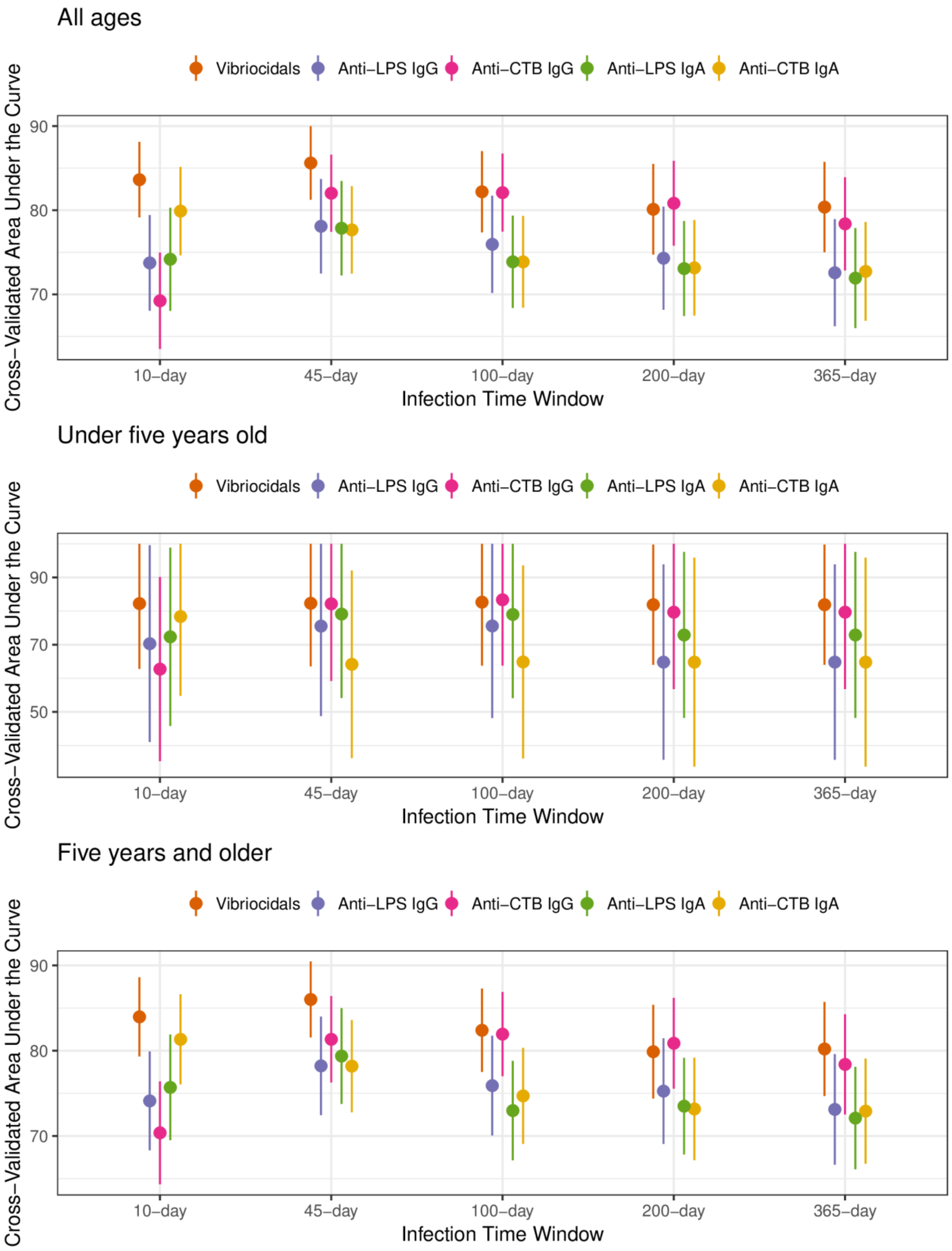

Fig. S7. cvAUC for each marker for different infection time windows by age group. Error bars represent the 95% confidence intervals. Note that there were too few data points in the IgM subset to include in these stratified analyses. Top panel is the same as Figure 3 in the main text and is duplicated here to allow for easier comparisons.

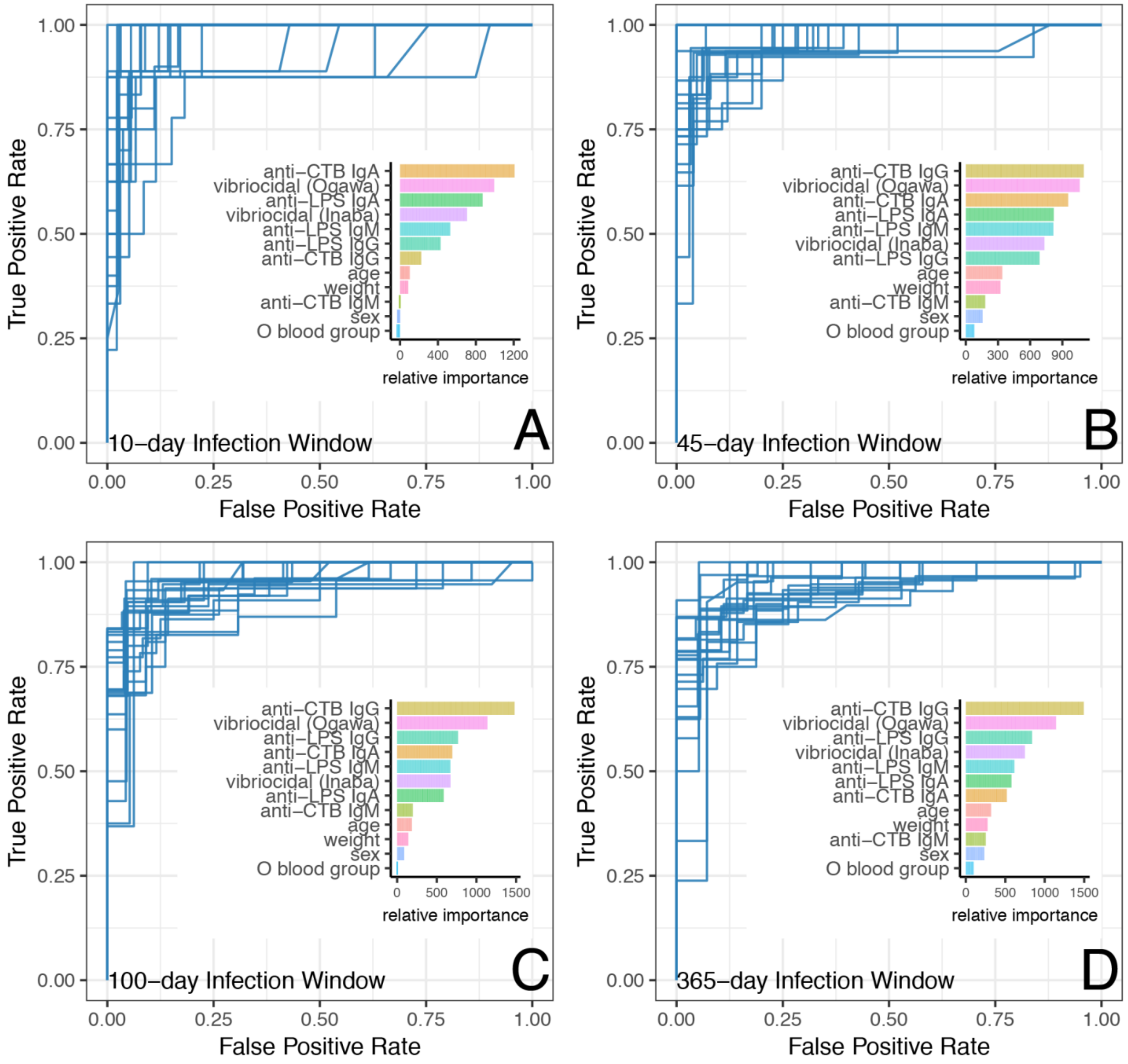

Fig. S8. cvAUC and variable importance from random forest models fit to subset of Bangladesh data with IgM measurement by infection time window. Insets for each panel show the distribution of relative importance of each variable (median across cross-validation folds), with larger values representing parameters with more influence in the final model prediction as assessed through a permutation test procedure.

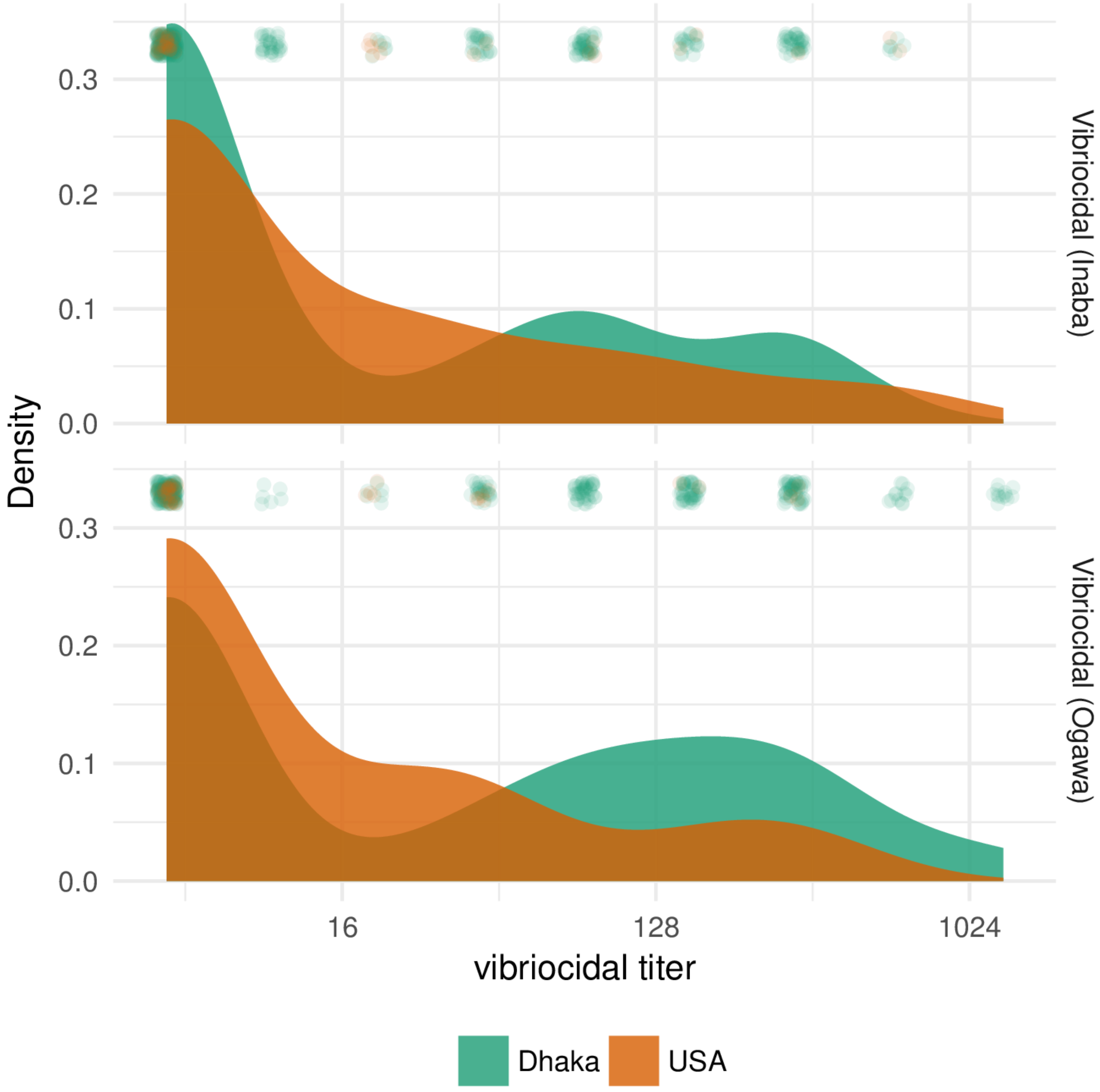

Fig. S9. Distribution of baseline vibriocidal titers in North American volunteers (United States, orange) and household contacts in Bangladesh (Dhaka, green). Colored dots illustrate data points (jittered) behind the density plot.

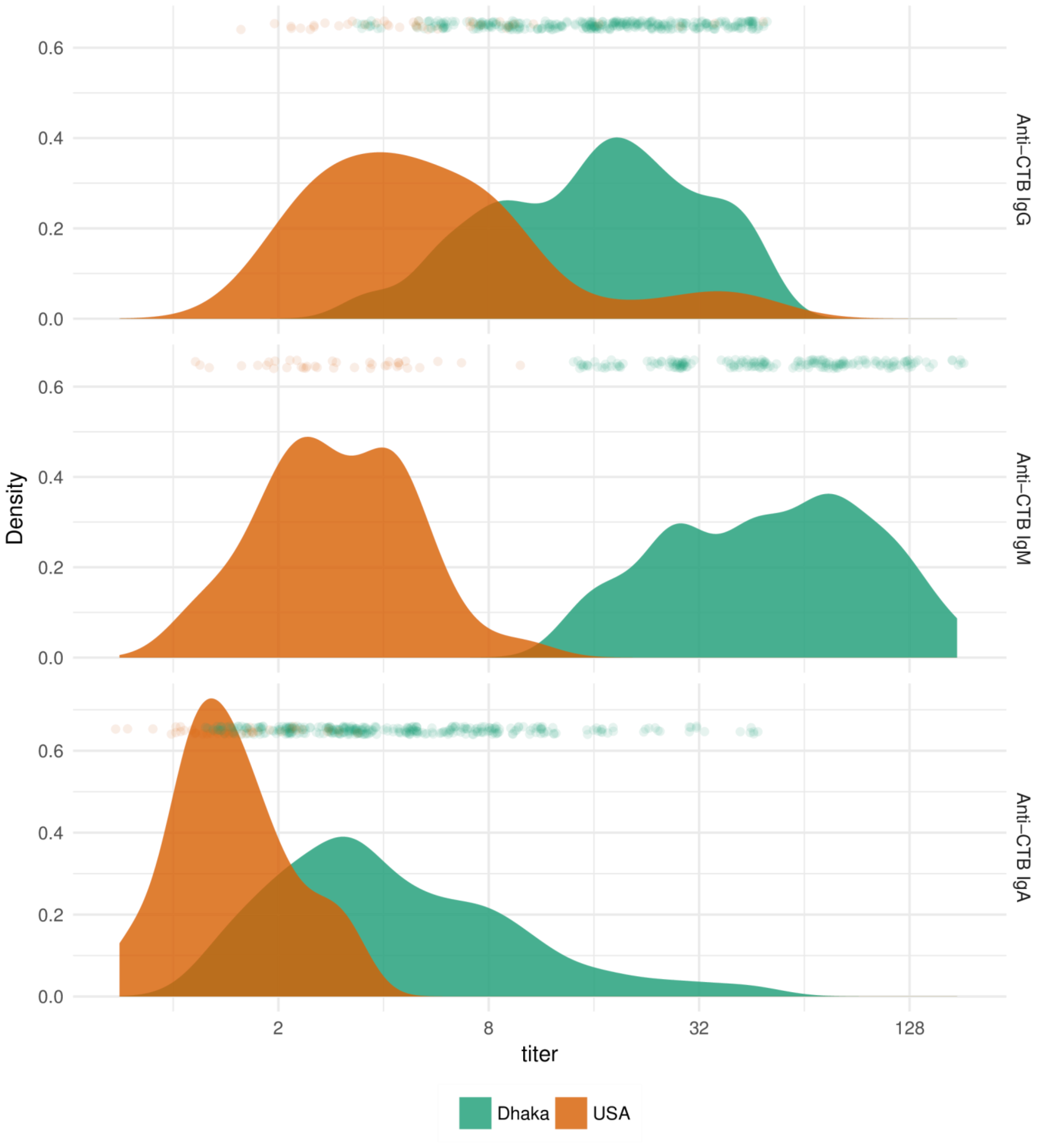

Fig. S10. Distribution of baseline anti-CTB IgG, IgM, and IgA titers in North American volunteers (United States, orange) and household contacts in Bangladesh (Dhaka, green). Colored dots illustrate data points (jittered) behind the density plot.

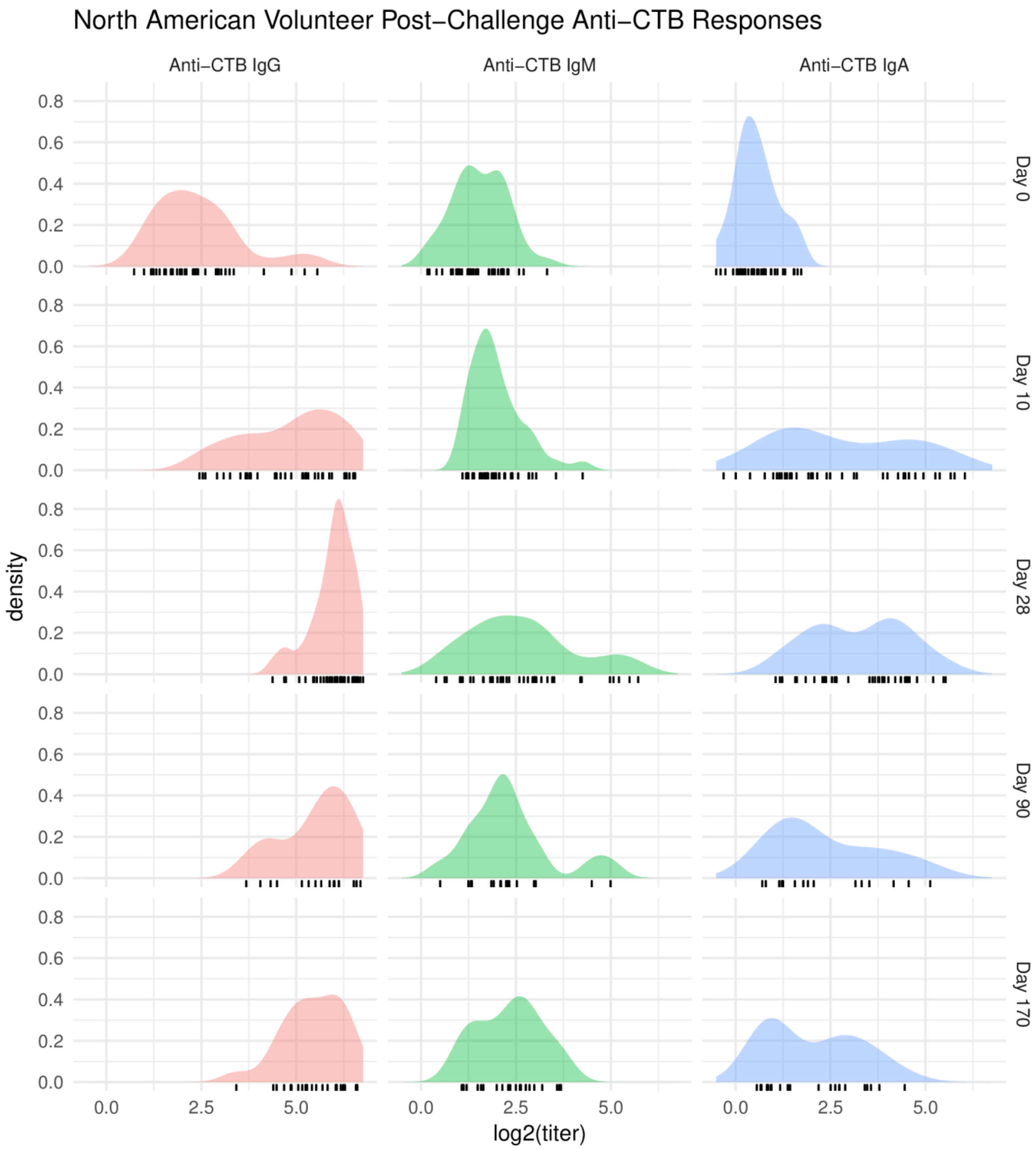

Fig. S11. Distribution of anti-CTM IgG (red), IgM (green), and IgA (blue) titers by day after experimental infection among North American volunteers. Exact data points (n=38) are shown under each curve in black.

Table S1. Differences in (log_2) median titer at baseline between subgroups for each marker.

	under 5	O blood group	sex
Anti-CTB IgA	0.29 (-0.42, 0.99)	0.21 (-0.03, 0.52)	-0.19 (-0.51, 0.07)
Anti-CTB IgG	0.75 (0.39, 1.12)	-0.07 (-0.32, 0.24)	-0.35 (-0.65, 0.01)
Anti-LPS IgA	-0.68 (-1.15, -0.20)	0.19 (-0.24, 0.53)	0.07 (-0.43, 0.44)
Anti-LPS IgG	0.20 (-0.20, 0.59)	-0.09 (-0.31, 0.18)	-0.17 (-0.47, 0.05)
Anti-LPS IgM	0.29 (-0.22, 0.81)	0.10 (-0.39, 0.36)	-0.19 (-0.45, 0.22)
Inaba Vibriocidal	-1.22 (-2.13, -0.31)	0.00 (-1.00, 0.00)	-0.50 (-2.00, 0.00)
Ogawa Vibriocidal	-0.65 (-1.62, 0.31)	0.00 (-1.00, 0.00)	0.00 (-1.00, 1.00)

Table S2. cvAUC for single markers within the Bangladesh cohort over different infection time windows. Note that anti-LPS and anti-CTB IgM were only available for 202 participants. 95% confidence intervals are shown in parentheses.

Time Window	Vibriocidal	Anti-CTB IgG	Anti-CTB IgA	Anti-LPS IgG	Anti-LPS IgA	Anti-LPS IgM	Anti-CTB IgM
**10 day**	83.6% (79.1-88.1)	69.2% (63.5-75)	79.9% (74.7-85.1)	73.7% (68.1-79.4)	74.2% (68.1-80.3)	76.2% (66.1-86.3)	51.3% (45.5-57.0)
**45 day**	85.6% (81.2-90.0)	82% (77.4-86.6)	77.7% (72.5-82.9)	78.1% (72.5-83.7)	77.9% (72.3-83.5)	78.6% (68.9-88.4)	51.7% (46.2-57.2)
**100 day**	82.2% (77.4-87)	82.1% (77.5-86.7)	73.9% (68.4-79.3)	75.9% (70.2-81.7)	73.9% (68.4-79.4)	75.6% (67.2-84.0)	50.2% (44.6-55.7)
**200 day**	80.1% (74.7-85.5)	80.8% (75.8-85.9)	73.2% (67.5-78.8)	74.3% (68.2-80.4)	73.1% (67.4-78.7)	75.8% (66.7-84.9)	52.4% (46.6-58.3)
**365 day**	80.4% (75.0-85.7)	78.4% (72.9-83.9)	72.7% (66.9-78.6)	72.6% (66.2-78.9)	71.9% (66.0-77.9)	75.8% (66.7-85.0)	52.4% (46.6-58.3)

Table S3. Thresholds (modal titer) and cross-validated sensitivity and specificity for single-antibody threshold tests within the Bangladesh cohort by infection time window.

Marker	Time Window	sensitivity	specificity	modal titer
**CTB IgA**	10 days	87.1% (73.7-100.0)	77.0% (67.9-85.1)	14
	45 days	72.5% (60.0-84.8)	85.8% (77.3-93.0)	13
	100 days	77.6% (65.5-88.1)	76.3% (65.4-86.3)	9
	200 days	73.2% (62.3-83.6)	79.0% (67.4-89.3)	9
	365 days	70.9% (60.3-81.7)	79.7% (68.2-90.0)	9
**CTB IgG**	10 days	81.7% (64.0-95.5)	61.9% (51.7-71.6)	37
	45 days	86.1% (75.5-94.7)	78.7% (69.0-88.2)	37
	100 days	81.9% (72.2-91.1)	84.7% (75.4-93.0)	37
	200 days	74.9% (64.0-84.6)	88.7% (80.9-96.1)	38
	365 days	71.5% (61.3-81.4)	88.8% (79.5-97.0)	38
**CTB IgM**	10 days	82.0% (60.0-100.0)	28.6% (17.4-40.8)	44
	45 days	67.0% (46.7-85.2)	39.8% (25.0-54.1)	52
	100 days	66.4% (50.0-82.1)	41.1% (25.0-57.6)	52
	200 days	66.2% (50.0-81.1)	42.4% (25.0-60.7)	52
	365 days	66.5% (51.5-81.1)	42.1% (24.1-60.0)	52
**LPS IgA**	10 days	74.0% (55.0-90.5)	74.8% (65.6-83.3)	22
	45 days	67.3% (53.7-79.5)	88.4% (81.5-95.3)	23
	100 days	73.8% (62.0-84.3)	77.8% (67.7-87.7)	15
	200 days	69.0% (58.2-79.7)	80.1% (69.6-90.4)	15
	365 days	67.3% (56.1-77.8)	80.2% (68.9-90.5)	15
**LPS IgG**	10 days	71.4% (53.8-88.0)	76.7% (68.5-84.3)	65
	45 days	70.1% (57.8-81.8)	87.6% (79.1-95.0)	61
	100 days	64.7% (51.9-76.5)	90.7% (82.8-98.0)	61
	200 days	60.1% (47.8-71.9)	93.0% (86.0-98.3)	61
	365 days	57.8% (46.4-69.4)	93.4% (86.0-100.0)	61
**LPS IgM**	10 days	82.9% (60.0-100.0)	75.6% (63.5-86.7)	78
	45 days	75.0% (56.2-91.3)	86.3% (75.0-95.5)	78
	100 days	70.3% (52.4-85.7)	85.8% (73.5-96.8)	71
	200 days	64.2% (48.4-79.3)	88.1% (75.0-100.0)	71
	365 days	64.0% (48.4-79.3)	88.4% (75.8-100.0)	71
**Vibriocidal**	10 days	84.9% (70.0-96.3)	83.0% (75.9-90.1)	1280
	45 days	84.3% (74.0-94.0)	88.4% (80.6-95.2)	640
	100 days	76.4% (65.4-86.3)	91.4% (84.7-97.1)	640
	200 days	82.4% (72.7-91.0)	81.0% (70.2-90.6)	320
	365 days	80.6% (70.8-89.6)	83.0% (73.0-92.1)	320

Table S4. cvAUC from random forest models fit to IgM subset of Bangladesh data by infection time window. The full model includes all markers and demographics (same as those shown in Figure 4 subpanels), the two-marker model uses only the top two markers (for each window) from the full random forest model and the ELISA-only model uses anti-CTB and anti-LPS IgA and IgG titers. Vibriocidal Ogawa titers were used in all two-marker models; however, the 10-day model used anti-CTB IgA and the others used anti-CTB IgG. 95% confidence intervals are shown in parentheses.

	Infection Time Window				
Model	10 days	45 days	100 days	200 days	365 days
full model	95.9 (94.1-97.6)	96.8 (95.8-97.9)	94.7 (93.5-95.9)	94.9 (93.5-96.4)	94.7 (93.2-96.2)
two-marker	92.7 (89.4-95.9)	93.6 (91.5-95.7)	93.6 (92.2-95)	92.9 (91.1-94.6)	92.5 (90.7-94.2)
ELISA only	92.8 (90.6-95)	94.8 (93.3-96.3)	92.8 (91.3-94.3)	92.8 (91.1-94.6)	92.5 (90.7-94.3)

Table S5. cvAUC from random forest models fit to Bangladesh data subset of blood type O negatives only. The full model includes all markers and demographics (same as those shown in Figure 4 subpanels), the two-marker model uses only the top two markers (for each window) from the full random forest model and the ELISA-only model uses anti-CTB and anti-LPS IgA and IgG titers. Vibriocidal Ogawa titers were used in all two-marker models; however, the 10-day model used anti-CTB IgA and the others used anti-CTB IgG. 95% confidence intervals are shown in parentheses.

	Infection Time Window				
Model	10-days	45-days	100-days	200-days	365-days
full model	95 (93.3-96.6)	97.3 (96.4-98.3)	94.8 (93.5-96)	93.1 (91.4-94.8)	93.3 (91.5-95)
two-marker	90.8 (87.8-93.8)	94.4 (92.5-96.2)	93 (91.3-94.8)	90.2 (87.9-92.5)	89.6 (87.1-92.1)
ELISA only	90.7 (88.3-93.2)	93.4 (91.7-95.1)	91.2 (89.6-92.8)	88.3 (86.4-90.2)	86.5 (84.2-88.7)

Table S6. cvAUC from random forest models discriminating between infections occurring in different time windows. The rows represent the infection window for those classified as 'recently infected' with the comparison group indicated by the columns. The top left entry, for example, shows that saturated (all markers) random models had an AUC of 88.5% for discriminating between infections that occurred 5-10 days before compared to those that occurred 10-45 days in the past. 95% confidence intervals are shown in parentheses.

	Comparison Group Infection Window			
Case Group Infection Window	10-45 days	45-100 days	100-200 days	200-365 days
5-10 days	88.5 (86.3-90.8)	96.6 (95.1-98.1)	97.0 (95.6-98.5)	97.8 (96.3-99.4)
10-45 days	-	90.3 (87.7-92.9)	95.5 (93.6-97.3)	96.9 (94.4-99.4)
45-100 days	-	-	65.9 (60.8-71.0)	69.5 (64.6-74.3)
100-200 days	-	-	-	66.6 (59.6-73.7)

Table S7. Sensitivity and specificity of single-antibody thresholds in an external validation set of North American volunteers (*n* = 38) over different time windows.

Marker	Time Window	Sensitivity	Specificity
Vibriocidal	45	97.4	86.8
	100	85.9	91.7
	200	85.1	94.7
Anti-CTB IgG	45	69.7	64.5
	100	69.6	73.3
	200	68.4	94.7
Anti-CTB IgA	45	40.8	93.4
	100	43.5	91.7
	200	42.1	100

Table S8. Estimated mean annual incidence, MAE, and mean bias error from both random forest models and corrected vibriocidal tests. Estimates are based on 1,000 simulated endemic years and serosurveys with sample sizes of 500, 1,000 and 3,000 in a population of 25,000. Mean bias is estimated as the mean difference between the 'true' and estimated incidence over all simulated serosurveys.

		Random Forest			Vibriocidal, Corrected*		
Sample Size	Annual Incidence per 1,000	Mean Incidence	Mean Absolute Error (%)	Mean Bias (%)	Mean Incidence	Mean Absolute Error (%)	Mean Bias (%)
500	2.0	2.2	76.7	12.0	8.4	438.0	318.8
	5.0	5.6	53.9	12.5	9.4	203.9	88.2
	10.0	11.2	37.6	12.3	11.9	127.1	19.2
	50.0	56.1	18.8	12.1	38.3	46.8	-23.4
	100.0	112.1	14.9	12.1	80.0	28.5	-20.0
1000	2.0	2.2	56.2	11.8	5.4	293.3	169.0
	5.0	5.6	37.2	12.0	6.4	148.5	29.0
	10.0	11.2	27.1	12.1	9.0	98.7	-10.3
	50.0	56.1	15.1	12.1	37.3	37.5	-25.5
	100.0	112.0	13.0	12.0	79.9	23.8	-20.1
3000	2.0	2.2	33.3	12.0	2.5	164.3	27.2
	5.0	5.6	22.6	12.0	3.3	99.3	-34.6
	10.0	11.2	17.6	12.1	5.9	74.2	-41.2
	50.0	56.0	12.5	12.1	37.2	28.5	-25.7
	100.0	112.1	12.2	12.1	80.1	20.4	-19.9

*when corrected number of positive individuals was <0, they were assigned the value 0.

Table S9. cvAUC for random forest models trained on a subset of data (*n* = 347) excluding potentially infected household contacts. Household contacts excluded were those assigned to the upper distribution (of titers) in the two-component mixture model described in the main text.

	Infection Time Window				
Model	10-days	45-days	100-days	200-days	365-days
full model	94.3 (92.8-95.8)	96.9 (96-97.8)	95.1 (94.2-96.1)	94.5 (93.3-95.6)	94.4 (93.2-95.6)
two-marker	91.7 (89.5-93.9)	94.5 (93.1-95.8)	93.9 (92.7-95.2)	93.2 (92-94.4)	92.9 (91.5-94.4)
ELISA only	89.9 (87.8-92)	93.6 (92.3-94.9)	92 (90.7-93.3)	90.1 (88.7-91.6)	87.8 (86-89.6)
